# Experimental dipole moment prediction with a progressive 2D–3D hybrid framework and twin-pair-based diagnostic evaluation

**DOI:** 10.1186/s13321-026-01215-4

**Published:** 2026-06-14

**Authors:** Sadettin Y. Ugurlu, Shan He

**Affiliations:** 1 Novexus Ltd, Antalya, 07058 Turkey; 2 Department of Materials Science and Engineering, Faculty of Engineering, Akdeniz University, Dumlupinar Bulvari, Antalya, 07058 Turkey; 3 School of Computer Science, University of Birmingham, Edgbaston, Birmingham, B15 2TT United Kingdom

**Keywords:** Dipole moment prediction, Twin-pair analysis, MACCS keys, Hybrid ensemble learning, 3D graph neural networks (EGNN), Avalon fingerprints (radius-controlled)

## Abstract

**Abstract:**

Accurate prediction of molecular dipole moments (μ) is essential for modeling electrostatic interactions in solvation, molecular recognition, and materials. While modern 3D learning models can approach saturation on low-noise quantum-chemical benchmarks, performance often degrades on experimentally compiled datasets where measurement noise, stereochemical ambiguity, and conformer variability introduce a substantial gap between idealized theory and practical data. Here, we present a progressive 2D–3D hybrid framework for experimental dipole-moment prediction under heterogeneous labels. The framework is constructed in two stages. First, a strong 2D tabular CatBoost model based on SMILES-derived multi-view fingerprints is enriched with physicochemical, dipole-related, and conformer-dependent 3D shape descriptors, introducing a first level of 2D+3D integration and improving performance over the 2D representation alone. Second, this enriched tabular predictor is linearly fused with a geometry-aware 3D graph neural network operating on conformer-derived molecular graphs, yielding a further gain through complementary structural learning. On the experimental test set, the resulting framework achieves $R^{2}=0.844$ with $\textrm{MAE}=0.399~\textrm{D}$, outperforming a nonparametric fingerprint *k*-NN baseline ($R^{2}=0.65$) and remaining close to the similarity-conditioned diagnostic reference range estimated from twin-pair dispersion ($R^{2}\approx 0.86$–0.91) under Tanimoto similarity thresholds of 0.95, 0.97, 0.98, and 0.99.

**Scientific contribution:**

The main scientific contribution of this study is the development of an experimentally grounded progressive 2D–3D hybrid learning framework that integrates enriched tabular chemical representations, geometry-aware graph learning, and twin-pair-based diagnostic evaluation to predict dipole moments under heterogeneous experimental labels. To contextualize performance in this noise-affected experimental setting, we further use twin-pair analysis within a test-to-train similarity-stratified evaluation protocol, while baseline comparisons and component-wise interpretability analyses help clarify the roles of representation diversity and learner complementarity. Relative to three recently published dipole-prediction studies, the proposed framework remains competitively positioned, although these comparisons are intended only for cautious literature contextualization because the underlying datasets, label sources, and noise regimes are not directly matched. In contrast to prior studies focused mainly on lower-noise theoretical benchmarks or narrower molecular domains, the present work targets the comparatively underexplored Stenutz experimental compilation.

**Supplementary Information:**

The online version contains supplementary material available at 10.1186/s13321-026-01215-4.

## Introduction

The dipole moment (μ) is a fundamental electronic property that describes the magnitude and direction of charge separation within a molecule, reflecting the combined effects of molecular geometry and asymmetric electron distribution [1–4]. Because it governs intermolecular electrostatic interactions, μ is closely linked to key macroscopic phenomena such as solubility, phase behavior, and interaction energies [5–7]. Accordingly, reliable dipole-moment prediction remains important for understanding and modeling molecular behavior across chemical space.

The ability to predict dipole moments rapidly and reliably offers a strategic advantage in both materials science and medicinal chemistry [8]. In the domain of organic electronics and photonics–including organic photovoltaics (OPVs) [2], OLEDs [9], and functional organic semiconductors [10]–the dipole moment and associated electronic parameters are intrinsically linked to performance-determining processes, such as energy level alignment, charge transport, dielectric response, and interface dipoles [11–13]. In the context of drug discovery, μ functions as a proxy for polarity, correlating with solubility, membrane permeability, and target binding behavior, thereby indirectly influencing ADME-oriented outcomes, such as logP and bioavailability [14–18]. Therefore, the estimation of electronic properties, such as μ, during early-stage high-throughput screening (HTS) enhances the decision quality and development efficiency of QSPR/QSAR models by minimizing reliance on costly experimental-computational trial-and-error cycles [14, 19, 20].

Conventionally, dipole moments are obtained experimentally through spectroscopy or theoretically through quantum chemical methods such as Density Functional Theory (DFT) and ab initio approaches [18, 21–23]. Although these approaches can provide high accuracy, their practical use in large-scale chemical space exploration is limited by cost and scalability [24–26]. As a result, machine-learning-based alternatives have increasingly been explored for dipole-moment prediction across both general molecular datasets and more specialized quantum settings [27, 28]. However, converting molecular structure into a machine-readable representation remains challenging, and for μ several issues become especially critical: (i) experimentally compiled labels contain measurement-induced aleatoric uncertainty, (ii) 2D encodings derived from SMILES or fingerprints may obscure stereochemical distinctions and conflate related molecular forms, and (iii) 3D deep-learning pipelines often depend on generated single-conformer proxies that may not reflect the true conformer ensemble [29–33]. Consequently, performance achieved on low-noise theoretical benchmarks and recent ML-based dipole studies (e.g., $R^2>0.99$ and $\textrm{MAE}\approx 0.03~\textrm{D}$) [34, 35] often does not transfer directly to heterogeneous experimental compilations. The central challenge, therefore, is to develop a generalizable prediction framework that remains robust under experimental noise and representation-related uncertainty.

In this study, we propose a progressive 2D-3D hybrid framework for dipole moment (μ) prediction from heterogeneous experimental measurements, with the primary contribution centered on staged hybridization at both the representation and model levels. First, a strong tabular baseline is established using a CatBoost branch built on SMILES-derived multi-view fingerprints. This branch is then enriched with auxiliary physicochemical, dipole-related, and conformer-dependent 3D shape descriptors, yielding a first level of 2D+3D integration within the tabular model itself. Finally, a second and higher-level hybridization step is introduced by linearly fusing this enriched CatBoost predictor with a geometry-aware 3D GNN operating on conformer-derived molecular graphs. Taken together, these stages define a hierarchical fusion strategy in which progressively enriched tabular chemical priors are complemented by explicit graph-based structural learning, yielding improved generalization under heterogeneous experimental labels. To support interpretation in this challenging experimental setting, the study further introduces twin-pair analysis to quantify similarity-conditioned label dispersion. It uses it within a similarity-stratified evaluation framework to provide a regime-dependent diagnostic reference for performance interpretation rather than a strict physical upper bound. Additional support is provided through a nonparametric similarity baseline, component-wise ablation and complexity-ladder analyses, feature-level interpretability analyses, and a tautomer-aware sensitivity analysis examining whether canonical structure assignment may obscure tautomer-dependent variation relevant to experimentally measured dipole moments. Unlike prior dipole-prediction studies focused mainly on lower-noise theoretical benchmarks, the present work targets experimentally measured dipole moments in a substantially more heterogeneous and comparatively underexplored setting. Overall, the framework is intended not only as a predictive model, but also as a more interpretable and experimentally grounded strategy for understanding what can realistically be learned from experimental dipole-moment data.

The codes, trained model, and framework are freely available for academic use:

https://github.com/yauz3/dipole_moment

## Methods and materials

### Data collection and curation

The dataset comprising experimental dipole moments (μ, Debye) for organic compounds was curated from the publicly available repository provided by Stenutz.eu (https://www.stenutz.eu/chem/dipole.php). Importantly, while the Stenutz compilation can be viewed as a valuable experimental resource for dipole-moment modeling, it also represents a comparatively underexplored and structurally challenging benchmark. Unlike cleaner theoretical datasets built from explicitly optimized molecular geometries, this collection provides compound names that must be resolved into canonical structures before modeling, without direct access to experimentally matched 3D conformers, solvent- and temperature-dependent tautomer populations, or other environment-specific molecular states. Consequently, the benchmark contains not only real experimental signal but also substantial ambiguity arising from heterogeneous measurement conditions, solvent- and phase-related effects, temperature-dependent state variation, conformational mismatch, tautomeric uncertainty, and incomplete structural standardization. This combination makes the dataset especially demanding but also scientifically valuable because it enables machine-learning evaluation in a more realistic and less curated regime than conventional dipole-moment benchmarks.

Chemical structures were standardized at the level of one-dimensional representations by resolving compound names to canonical SMILES strings. Briefly, compound names were first normalized by removing leading stereochemical prefixes, transliterating Greek letters to alphabetic equivalents, and harmonizing common naming variants (e.g., converting ’α-pinene’ to ’alpha-pinene’). The normalized names were then queried against PubChem [36] using the PubChemPy [36, 37] interface to retrieve canonical SMILES strings. To ensure robustness, transient server-side errors (e.g., HTTP 503) were handled via an exponential backoff strategy with repeated attempts (up to five retries per compound). For compounds that did not return a valid PubChem record, the normalized names were subsequently queried against the NCI Cactus chemical structure service as an alternative resolver [38]. The resulting SMILES strings were integrated into the dataset as the primary structural input for downstream modeling.

### Feature extraction from SMILES

A multi-view SMILES-based fingerprint strategy was used to avoid dependence on a single 2D encoding. Each molecule was parsed with RDKit, and invalid SMILES were assigned all-zero vectors to preserve dataset alignment. Molecular structure was encoded using MACCS keys together with 16 radius-controlled Avalon configurations generated by varying radius (0-3) and bit length (128, 256, 512, 1024). This design was adopted to provide controlled representation diversity within the 2D branch rather than committing to a single fingerprint parameterization. MACCS keys serve as a compact and chemically interpretable structural baseline, while Avalon fingerprints provide a flexible hashed encoding with broader substructure coverage. By varying radius and bit length, the Avalon component captures molecular environments at multiple neighborhood scales and resolutions, reducing sensitivity to any single predefined encoding choice. The resulting combination was therefore used as the fixed multi-view 2D representation throughout the study.

To reduce the sensitivity of the geometry-dependent branch to an arbitrary single embedding, conformational sampling was performed before selecting the representative 3D structure. For each molecule, up to 20 candidate conformers were generated using the RDKit ETKDG/ETKDGv3 protocol, followed by MMFF94 relaxation when available, with UFF fallback disabled by default. The lowest-energy valid conformer was then retained as the representative geometry for graph construction. In parallel, the enriched CatBoost branch was extended with conformer-dependent auxiliary descriptors, including 7 dipole-related proxy variables and 10 3D shape descriptors, so that conformational information contributed not only through the 3D GNN branch but also through the tabular branch.

### Dataset size, split transparency, and reproducibility

After name normalization, SMILES resolution, and structural curation, the final dataset used for modeling contained 4,979 molecules with valid target values, of which 3,983 molecules were assigned to the training portion and 996 were reserved as the unseen test set. The study therefore followed a fixed 80/20 train-test partition, and the unseen test benchmark was kept completely separate from all stages of model development. All hyperparameter tuning, model selection, and internal optimization were performed exclusively within the training portion, using a dedicated internal training-validation split that was distinct from the folds later used in the main 5-fold cross-validation analysis. Thus, the validation subset used during tuning was not one of the reported evaluation folds, but a separate holdout partition created solely for model selection and hyperparameter search. After the optimal configuration had been identified on this internal validation split, the corresponding model was retrained on the merged in-sample data and then evaluated under the predefined benchmarking protocol.

To assess robustness under the same split design, the finalized models were further examined using 5-fold cross-validation within the training portion. In each iteration, the model was fitted on four folds and evaluated on the remaining fold, and the fold-trained model was additionally used to generate predictions on the fixed 20% unseen test set. This produced five independent test predictions, allowing final test performance to be summarized across folds rather than relying on a single training realization. This procedure was adopted to preserve a clean separation between hyperparameter selection and performance estimation, while also improving data efficiency and providing a more stable estimate of generalization under heterogeneous experimental noise.

To ensure transparency and eliminate ambiguity regarding data partitioning, the exact processed split files, together with the full preprocessing, feature-generation, training, and evaluation scripts, have been made publicly available in the project GitHub repository (https://github.com/yauz3/dipole_moment), allowing the complete train-test setup to be reproduced. Importantly, the dataset itself was not additionally filtered on the basis of downstream 3D conformer or graph-building success, in order to avoid introducing selection bias into the overall benchmark design. Instead, the same predefined split structure was preserved throughout, while the repository records the exact data instances successfully processed in each experimental branch. This makes both the benchmark definition and the branch-specific reproducibility fully explicit.

### CatBoost, 3D GNN, and hybrid model architecture

Within the hybrid framework, the core tabular branch was centered on CatBoost and evaluated under two progressively enriched configurations. The first used the combined Avalon and MACCS fingerprint space as a strong 2D baseline, whereas the second extended this representation with 9 basic 2D descriptors, 7 dipole-related proxy variables, and 10 conformer-dependent 3D shape descriptors, introducing a first level of 2D+3D integration within the tabular branch. In parallel, a geometry-aware 3D GNN was trained on conformer-derived molecular graphs to capture spatial information not fully accessible to fixed tabular encodings. On this basis, two 1:1 linear hybrid variants were examined: CatBoost (fingerprints) + 3D GNN, and CatBoost (fingerprints + extended descriptor set) + 3D GNN. This progressive architecture allowed the contribution of staged representation enrichment to be assessed directly, from a pure 2D baseline, to an enriched tabular 2D+3D model, and finally to a higher-level hybrid framework combining tabular and graph-based structural learning.

#### Tabular branch: CatBoost learner

CatBoost was adopted as the tabular branch of the proposed framework to provide a strong and noise-tolerant learner over heterogeneous molecular inputs. Two progressively enriched CatBoost configurations were evaluated. In the first configuration, the model was trained on the concatenated MACCS and Avalon fingerprint space, providing a robust 2D tabular baseline. This fingerprint set was selected to increase representation diversity in a controlled radius-dependent manner: MACCS keys provide a compact and interpretable structural baseline, whereas the 16 Avalon configurations broaden substructure coverage across multiple radius and bit-length settings rather than relying on a single fixed fingerprint parameterization.

In the second configuration, the same fingerprint representation was expanded with an auxiliary descriptor block consisting of 9 basic 2D descriptors, 7 dipole-related proxy variables, and 10 conformer-dependent 3D shape descriptors. This design allowed CatBoost to be evaluated not only as a fingerprint-based learner, but also as an enriched tabular model integrating complementary physicochemical and geometry-aware descriptors. Missing values in the auxiliary descriptor block were handled by median imputation before concatenation with the fingerprint features, whereas the fingerprint branch itself was used directly as a sparse tabular representation.

From a modeling perspective, CatBoost is well-suited to this setting because gradient-boosted decision trees can accommodate sparse, heterogeneous, and mixed-scale feature spaces while capturing non-linear effects and higher-order feature interactions without extensive preprocessing or feature scaling. In the present implementation, CatBoost was trained with a fixed regression objective (RMSE), tree depth of 7, and 4000 boosting iterations, while hyperparameter selection was performed only on the internal validation split defined within the training data, without using the unseen test set or the reported evaluation folds for tuning.

#### 3D geometry-aware branch: stabilized EGNN-Style 3D GNN

To incorporate explicit molecular geometry, a 3D graph neural network (GNN) was constructed from conformer-derived atomic coordinates generated with RDKit [39]. Each molecule was represented as a molecular graph in which nodes correspond to atoms and edges correspond to covalent bonds. Node features encoded atomic identity together with six local scalar descriptors: total degree, formal charge, aromaticity, ring membership, total valence, and total hydrogen count. Edge features encoded bond type/order, while Cartesian atomic coordinates were retained as explicit geometric inputs.

A robust conformer-generation protocol was adopted to obtain a single representative geometry per molecule. For each molecule, up to 20 candidate conformers were generated using the ETKDG/ETKDGv3 protocol, followed by MMFF94-based relaxation when available. The lowest-energy valid conformer was then retained as the representative structure for downstream graph construction. By default, the implementation was restricted to organic molecules and excluded problematic metal-containing structures in order to improve force-field robustness and graph-generation stability. This design provides a consistent 3D proxy while avoiding the substantially higher computational cost of explicit conformer-ensemble learning.

The resulting molecular graphs were processed using an EGNN-style message-passing architecture (Table 1). In the present study, the 3D branch does not introduce a new equivariant message-passing formalism; rather, it applies an EGNN-inspired design together with practical stabilization measures to improve numerical robustness during training. Atomic numbers were first embedded into a 64-dimensional learned embedding space and concatenated with the six scalar node descriptors, after which the combined node representation was projected into a hidden dimension of 128 (Table 1). The network then applied four successive equivariant message-passing layers. For an edge (j→i), the message update depended on the destination and source node states, squared interatomic distance $r_{ij}^{2}$, and bond-type edge attributes. Node states were updated through residual-style transformations followed by layer normalization, whereas coordinate updates were regularized through three stabilization steps: (i) per-graph coordinate centering before message passing, (ii) bounded coordinate-update gating via a tanh nonlinearity, and (iii) distance damping through division by $(r_{ij}^{2}+1)$. These modifications were introduced to reduce numerical pathologies such as exploding coordinate updates and non-finite activations while retaining the geometry-aware inductive bias of the model.

**Table 1:** Main architecture, conformer-generation, graph-construction, and optimization settings for the stabilized EGNN-style 3D GNN used as the geometry-aware branch of the hybrid framework

Category	Setting
Node embedding dimension	64
Number of scalar node features	6
Hidden dimension	128
Number of EGNN layers	4
Edge features	Bond type / bond order
Graph pooling	Mean pooling
Readout head	Two-layer MLP with SiLU activation
Conformer generator	RDKit ETKDG / ETKDGv3
Maximum number of conformers	20
Geometry optimization	MMFF94 (UFF fallback disabled)
Representative structure	Lowest-energy valid conformer
Chemical filtering	Organic-only by default; metals excluded
Optimizer	AdamW
Learning rate	$3 \times 10^{-4}$
Weight decay	$1 \times 10^{-5}$
Batch size	16
Maximum epochs	250
Early stopping patience	40
Gradient clipping	1.0
Target transformation	Training-set standardization (*z*-score)
Validation strategy	Internal validation split from training data only
Coordinate stabilization	Per-graph centering + tanh gating + $(r_{ij}^{2}+1)^{-1}$ damping
Node-state stabilization	Residual-style updates + layer normalization

After message passing, a global molecular representation was obtained through permutation-invariant mean pooling over node embeddings and passed to a regression readout head to predict the dipole moment. Model optimization was performed with AdamW, and target values were standardized using the training-set mean and standard deviation before optimization and transformed back to the original Debye scale for evaluation. Hyperparameter selection and early stopping were carried out only on an internal validation split derived from the training data, without using the unseen test set or the reported evaluation folds for tuning. The principal architectural and optimization settings of the 3D branch are summarized in Table 1 for reproducibility and technical transparency.

#### Hybrid ensemble modeling: linear fusion

To improve robustness under heterogeneous experimental noise and to exploit complementary inductive biases, a hybrid ensemble strategy was constructed by fusing two methodologically distinct learners: (i) a geometry-aware 3D GNN operating on conformer-derived molecular graphs, and (ii) a tuned CatBoost regressor operating on an enriched tabular molecular representation. In the CatBoost branch, two levels of tabular modeling were examined: a baseline fingerprint configuration based on Avalon + MACCS, and an expanded configuration that further incorporated 9 basic 2D descriptors, 7 dipole-related proxy variables, and 10 conformer-dependent 3D shape descriptors. This design allowed the tabular branch itself to absorb additional physicochemical and geometric information before external fusion with the 3D GNN.

The final hybrid model was defined through simple linear fusion with equal weights (1:1) between the optimized CatBoost predictor and the 3D GNN. This formulation was intentionally kept minimal and transparent in order to reduce model complexity, avoid additional bias arising from weight selection, and maintain focus on the central question of the study: how effectively a practically deployable 2D+3D framework can recover predictive signal under experimentally heterogeneous labels. Under this design, CatBoost contributes robust structure-derived priors from fingerprint and descriptor space, whereas the 3D GNN contributes geometry-sensitive structural information aligned with the vector nature of dipole moments. Their combination therefore provides a principled mechanism for averaging partially non-redundant error modes and improving generalization in a similarity-dependent experimental setting characterized by substantial label dispersion.

### Performance metrics

Predictive performance was quantified using three complementary regression metrics: the coefficient of determination ($R^{2}$), mean absolute error (MAE), and root mean squared error (RMSE). The $R^{2}$ statistic measures the fraction of variance in the target explained by the model, providing an interpretable assessment of global fit. MAE reports the average absolute deviation in the original units of the dipole moment ($\textrm{D}$), offering a robust, scale-preserving estimate of typical error magnitude. RMSE penalizes larger deviations more strongly than MAE, making it sensitive to occasional large errors and thus informative about tail-risk behavior. Using $R^{2}$, MAE, and RMSE jointly enables balanced evaluation across overall explanatory power, typical absolute accuracy, and sensitivity to high-error cases, which is particularly relevant for experimentally compiled datasets where heteroscedastic noise and outliers may occur.

### Comparison analysis

Two comparison settings were used: (i) an external literature-level comparison for contextual positioning and (ii) an internal controlled comparison across candidate models, representations, and hybrid variants under the same data split and validation protocol.

#### External comparative methodology across representative dipole-moment prediction paradigms

To position the proposed hybrid ensemble within the broader dipole-moment prediction literature, five representative studies [27, 28, 33–35] were selected to span complementary methodological and data-regime paradigms rather than to support strict like-for-like superiority claims. These references cover: (i) highly optimized 3D deep-learning models trained on large, near-noise-free quantum benchmarks, (ii) recent chemistry-informed graph learning frameworks built on DFT-derived targets, (iii) classical descriptor-driven machine learning for dipole prediction, and (iv) low-data dipole-learning settings defined over narrower molecular domains. Accordingly, SchNet-class continuous-filter architectures provide a canonical high-performance reference under QM9-style geometry and label conditions [34]. Recent pretrained E(3)-equivariant message-passing models represent the current frontier of geometry-aware molecular learning when high-fidelity quantum-derived labels are available [35]. Q-DFTNet further reinforces the continued relevance of QM9/DFT-centered dipole benchmarking through a chemistry-informed graph-learning formulation [27]. As a chemically narrower but still informative comparison, the diatomic-molecule study of Elhalawani et al. [28] illustrates dipole prediction in a low-data setting over a substantially different molecular domain [28]. Finally, the descriptor-based Random Forest study by Pereira et al. provides a useful classical reference in terms of dipole-property focus and machine-learning philosophy [33]. These studies [27, 28, 33–35] serve primarily as reference points for positioning the proposed hybrid framework, rather than as directly commensurate benchmarks, since their target regimes, molecular domains, and underlying noise structures differ substantially from those of the present study.

The current study is centered on the Stenutz experimental dipole compilation, which represents a valuable real-measurement resource but also defines a substantially harder experimental setting than curated DFT benchmarks. In contrast to QM9-style targets, experimentally compiled dipole moments are affected by measurement variability, conformer dependence, stereochemical ambiguity, heterogeneous provenance, and residual reporting inconsistencies. The relative scarcity of prior machine-learning studies built directly on this compilation further reflects the challenge of this setting. Accordingly, the selected external studies are used to define the methodological neighborhood of the present work and to clarify its position within the dipole-prediction literature, rather than to support direct cross-dataset claims of absolute superiority [27, 28, 33–35].

As a nonparametric anchor for the experimental setting, an Experimental k-NN baseline (k=5) was included as a similarity-driven lower reference. Because this baseline uses the same broad fingerprint family as the 2D branch of the framework but relies only on nearest-neighbor transfer, it provides a stringent test of whether the learned models extract signal beyond local structural similarity alone.

To contextualize the achievable performance range under experimental uncertainty, a twin-pair-derived diagnostic reference was also estimated using twin-pair dispersion analysis. This reference reflects dataset-specific label consistency rather than a physically rigorous upper bound, and is used here to interpret model performance more appropriately under heterogeneous experimental labels.

Taken together, this comparison design serves three purposes: it situates the proposed framework within representative dipole-prediction paradigms, anchors the Stenutz experimental setting through explicit lower and diagnostic references, and clarifies that the aim is not direct outperformance of models trained on fundamentally different datasets, but robust prediction under a substantially harder and experimentally heterogeneous setting.

#### Intra-framework performance assessment

*Low-complexity baselines and complexity ladder:* To assess whether the final framework complexity was justified, additional low- to moderate-complexity baselines were evaluated under the same predefined train-test split. These included simple 2D descriptor/fingerprint models, dipole-proxy and 3D-shape descriptor sets, and fused 2D+3D tabular variants, trained with conventional regressors such as linear models, k-NN, tree ensembles, SVR-RBF, and CatBoost.

*Core framework component and hybridization analysis:* An intra-framework comparison was performed under the same predefined 80/20 train-test split and 5-fold cross-validation protocol. CatBoost, the stabilized EGNN-style 3D GNN, feature-view ablations, and linear hybrid variants were evaluated under identical conditions. Performance was summarized using $R^2$, MAE, and RMSE; fold-wise mean ± standard deviation is reported where cross-validation summaries are presented, whereas some analyses are reported as single held-out test-set summaries.

*Tautomer-aware sensitivity analysis under canonical and tautomer-expanded molecular-state construction:* To examine whether canonical structure assignment may obscure tautomer-dependent variation relevant to experimental dipole moments, an additional tautomer-aware sensitivity analysis was performed using the 3D GNN branch. In contrast to the main conformer-based pipeline, which operates on a single resolved molecular structure per compound, this auxiliary experiment explicitly expanded each compound into multiple plausible tautomeric states prior to 3D graph construction.

Because the source dataset did not provide experimentally resolved tautomer-specific 3D structures, the procedure began from a parent molecular representation defined at the identifier level. When a curated SMILES string was already available, it was used directly; otherwise, the parent structure was resolved from the compound name. This parent representation was then converted into a canonical molecular graph for downstream canonical-only or tautomer-expanded processing. From this parent structure, a bounded set of plausible tautomers was enumerated using a rule-based tautomer standardization framework. To avoid uncontrolled combinatorial growth, the number of retained tautomeric states per molecule was capped at a fixed maximum, and duplicate tautomeric forms were removed after canonicalization.

For each retained tautomer, a 3D conformer generation procedure based on RDKit was applied. Hydrogen-completed molecular structures were embedded in three dimensions using an ETKDG-style geometry generation protocol, followed by multi-conformer sampling and MMFF-based energy minimization. A fixed number of conformers was generated for each tautomeric state, and the lowest-energy conformer was retained as the representative geometry for graph construction. Each resulting 3D structure was then converted into a molecular graph in which nodes encoded atomic identity and basic atom-level scalar descriptors, while edges encoded bonded connectivity and bond-type information. Cartesian coordinates from the optimized conformer were preserved as geometric inputs.

The graph-regression architecture followed the same general stabilized EGNN-style design used in the main 3D GNN experiments, while the present sensitivity analysis employed separately constructed canonical-only and tautomer-aware datasets, each trained with its own internal training-validation split. In brief, atom types were embedded, concatenated with scalar atom descriptors, and processed through multiple equivariant message-passing layers. These layers used edge-wise messages dependent on node embeddings, squared interatomic distances, and bond features, together with bounded coordinate updates and graph-wise coordinate centering for numerical stability. Graph-level embeddings were obtained by mean pooling over node representations and mapped to scalar dipole predictions through a regression head.

Two parallel settings were evaluated on the same predefined train/test partition. In the canonical-only setting, each molecule contributed a single resolved parent structure represented by one optimized 3D state. In the tautomer-aware setting, each molecule contributed multiple tautomer-specific graphs, and the final molecular prediction was obtained by arithmetic mean pooling across tautomer-state embeddings. Thus, the comparison isolated the effect of tautomer-state expansion while keeping the downstream graph-learning framework otherwise unchanged.

Model training was performed separately for the canonical-only and tautomer-aware datasets. In each case, the training portion was further split into training and validation subsets, target values were standardized using the training-set mean and standard deviation, and optimization was carried out with AdamW. Early stopping was applied based on validation MAE. Predictive performance on the unseen test set was quantified using $R^2$, mean absolute error (MAE), and root mean squared error (RMSE). This experiment was designed as a sensitivity analysis rather than a full tautomer-resolved reconstruction of the experimental labels, since solvent-specific and temperature-specific tautomer populations were not available in the source compilation.

## Results and discussion

The results are discussed within a progressive 2D-3D framework for experimental dipole-moment (μ) prediction. The modeling strategy advances from a fingerprint-based CatBoost baseline to an enriched tabular 2D+3D representation and finally to linear fusion with a geometry-aware 3D GNN. To interpret performance under heterogeneous experimental labels, twin-pair analysis is used to quantify similarity-conditioned label dispersion and to provide a diagnostic reference for contextualizing predictive gains. This structure allows the contribution of staged representation enrichment and higher-level hybridization to be assessed under a realistic experimental setting.

### Comparative analysis

To position the proposed hybrid ensemble both within the broader dipole-moment prediction literature and within its own controlled design space, two complementary comparative analyses are reported. First, (i) Cross-Study Comparative Analysis benchmarks the framework against representative state-of-the-art studies while explicitly acknowledging differences in label regime (noise-free theoretical/DFT targets versus noisy experimental measurements), chemical diversity, and input representations. Second, (ii) Internal Framework Benchmarking and Performance Analysis provides a like-for-like evaluation of a broad set of baseline regressors and targeted ablations under an identical experimental protocol, enabling systematic attribution of performance differences to representation choice, model class, and hybrid fusion strategy.

#### Cross-study comparative analysis

At the broadest level, the high-performance QM9-based studies of Schütt et al. [34] and Xu et al. [35] are used here primarily for methodological contextualization rather than for direct benchmark-level comparison, as they demonstrate that dipole-moment prediction can approach near-error-free performance when the learning problem is defined on highly standardized, low-noise theoretical data with optimized geometries and internally consistent quantum-chemical labels. In this sense, these studies show that dipole moments can, in principle, be learned with extremely high accuracy, including the regime of $R^2>0.99$, when the dataset and structural inputs are sufficiently clean. Relative to that setting, the present hybrid framework operates under a substantially harsher experimental regime; therefore, its value lies not in matching QM9-level saturation, but in recovering robust predictive signal under heterogeneous real-world labels.

Pereira et al. [33] provide a useful theoretical reference from a different angle, reporting $R^2=0.87$ with $\textrm{MAE}=0.44~\textrm{D}$ using classical machine learning on DFT-derived dipole moments (Table 2). Although this is still a cleaner theoretical setting than the one used here, it shows that strong dipole-moment prediction is also achievable outside the near-saturation deep-learning regime. In that context, the present hybrid model, with $R^2=0.84$ and $\textrm{MAE}=0.40~\textrm{D}$ on experimentally compiled data, remains encouraging, especially because it reaches a competitive absolute error under a materially noisier and less standardized benchmark.

A further illustration of regime dependence is provided by Q-DFTNet [27], which also addresses dipole-moment prediction on QM9/DFT data. As summarized in Table 2, Wayo et al. reported $R^2=0.65$ with $\textrm{MAE}=0.62~\textrm{D}$ using a chemistry-informed GNN on this theoretical benchmark [27]. This highlights that even within the same broad QM9/DFT family, predictive performance still depends strongly on architecture, molecular representation, and evaluation setup, rather than being determined by the target property alone. In comparison, the current hybrid model proposed in this study achieves $R^2=0.84$ with $\textrm{MAE}=0.40~\textrm{D}$ on the heterogeneous experimental Stenutz benchmark (Table 2), indicating that a staged hybrid strategy combining enriched tabular 2D+3D priors with explicit 3D graph learning can remain practically effective even under a substantially noisier and less standardized prediction regime.

The study of Elhalawani et al. [28] offers a useful contrast from a much narrower molecular domain, reporting $\textrm{MAE}=0.45~\textrm{D}$ for diatomic molecules using atomic-property-based modeling (Table 2). Although this result is not directly comparable to broad small-molecule prediction, it reinforces that absolute error values must always be interpreted together with molecular-domain scope and structural complexity. Seen from this perspective, the performance of the current hybrid model, which achieves $\textrm{MAE}=0.40~\textrm{D}$ on a chemically broader and substantially more heterogeneous experimental dataset, remains encouraging.

To make cross-study interpretation meaningful under noisy experimental labels, we further anchor the comparison with two internal reference points defined directly within the Stenutz setting: (i) an experimental *k*-NN baseline (k=5), which serves as a stringent similarity-driven lower reference and attains only $R^2=0.65$ with $\textrm{MAE}=0.64~\textrm{D}$, and (ii) a twin-pair-derived diagnostic reference, which summarizes similarity-conditioned label dispersion under measurement variability and yields approximately $R^2 \approx 0.90$ with $\textrm{MAE} \approx 0.28~\textrm{D}$ (Table 2). Against these anchors, the proposed linear 1:1 hybrid fusion (CatBoost + 3D GNN) attains $R^2 = 0.84$ with $\textrm{MAE} = 0.40~\textrm{D}$, substantially outperforming the similarity baseline and remaining close to the twin-pair-derived diagnostic reference range. This supports the interpretation that the hybrid model captures non-redundant signal beyond local similarity alone, while still remaining appropriately constrained by the difficulty of the experimental regime.

**Table 2:** Comparative performance of the proposed framework, representative literature models, and internal baselines

Study	Methodology	Data source	Sample diversity	Input type	$R^2$	MAE	Key Insight
Pereira et al. [33]	Random Forest	Theoretical (DFT)	Moderate (DFT-optimized; cleaner labels)	2D descriptors	0.87	0.44	Classical ML on cleaner DFT targets; the error profile is not directly comparable to noisy experimental measurements.
Wayo et al. [27]	Chemistry-informed GNN (Q-DFTNet)	Theoretical (QM9/DFT)	Low (small organic molecules; curated benchmark)	Graph-based 2D molecular features	0.65	0.62	Recent chemistry-informed dipole-learning framework on DFT-driven QM9 data; useful for positioning within modern graph-learning literature, but still not directly comparable to noisy experimental labels.
Elhalawani et al. [28]	Gaussian Process Regression + symbolic regression	Mixed (experimental + high-level theory)	Very low (diatomic-only; narrow chemical domain)	Atomic properties only	–	0.45	Shows that recent dipole ML can also succeed in a low-data setting, but on a much narrower molecular space than heterogeneous small-molecule experimental dipole prediction.
Baseline	k-NN (k=5)	Experimental	High (chemically diverse; includes stereoisomers)	2D fingerprint	0.65	0.64	Similarity limit: nearest-neighbor similarity alone fails to capture geometry-driven vector cancellation and electronic effects.
Twin-pair reference	Intrinsic noise (“twin-pair”) analysis	Experimental	High (chemically diverse; includes stereoisomers)	Twin pairs via fingerprint similarity	≈ 0.90	≈ 0.28	Dispersion-based diagnostic reference derived from label variation among highly similar molecules; not a strict physical reference value.
3D GNN	Geometry-aware GNN	Experimental	High (chemically diverse; includes stereoisomers)	3D coordinates (generated conformers)	0.79	0.43	Gains from encoding 3D geometry; still constrained by conformer uncertainty and experimental noise.
CatBoost	Gradient boosting (CatBoost)	Experimental	High (chemically diverse; includes stereoisomers)	Enriched tabular 2D+3D	0.82	0.45	Strong tabular learner for high-dimensional descriptors/fingerprints; robust improvements over the k-NN baseline.
Hybrid (linear)	Linear fusion ensemble: 3D GNN + CatBoost	Experimental	High (chemically diverse; includes stereoisomers)	Tabular 2D+3D + 3D graph	0.84	0.40	Complementarity: 2D models capture broad substructure patterns while the 3D GNN addresses geometry-sensitive cases; fusion places performance close to the twin-pair-derived diagnostic reference range.

Studies are grouped by data source (theoretical vs experimental). Performance is reported as $R^2$ and MAE (Debye). For compact presentation, the *Input Type* labels are abbreviated: *CatBoost* refers to the enriched tabular 2D+3D representation, and *Hybrid (linear)* refers to its equal-weight fusion with the conformer-derived 3D GNN. Baseline denotes the similarity-based lower reference, and diagnostic reference denotes a noise-informed, similarity-conditioned diagnostic range rather than a strict physical upper bound

Taken together, these studies are best interpreted as reference points rather than as members of a single strict ranking, because the attainable performance in dipole-moment prediction is strongly regime-dependent. The literature shows that extremely high accuracy is possible on standardized theoretical datasets, while broader or noisier settings impose a much harder learning problem. Under that interpretation, the current hybrid framework appears promising: it does not aim to reproduce the easiest theoretical regime, but instead delivers strong and practically meaningful performance on a substantially more heterogeneous and unstandardized experimental dataset that has not yet been widely explored as a primary benchmark for ML-based dipole-moment prediction.

*Dataset regime and benchmark context:* To better contextualize the external comparison, it is important to distinguish not only the model class but also the dataset regime. The present study is based on the Stenutz experimental dipole compilation, which represents a comparatively underexplored benchmark characterized by heterogeneous real-measurement labels, name-resolved molecular structures, and substantial structural-condition ambiguity. By contrast, the representative literature considered here spans several markedly different regimes. Pereira et al. [33] used a DFT-based dataset of 10,071 organic molecules derived from ZINC and GDB-13, with descriptors computed from DFT-optimized geometries and partial atomic charges. Wayo et al. [27] trained and evaluated graph neural networks on the QM9 benchmark, i.e., a large-scale theoretical dataset built on quantum-chemically optimized small organic molecules and associated DFT-derived properties, following the broader QM9/GDB-9 data regime established by Ramakrishnan et al. [40]. In contrast, Elhalawani et al. [28] addressed a much narrower diatomic-molecule setting using atomic-property-based descriptors rather than a broad organic experimental benchmark. As summarized in Table 3, these studies are informative for methodological positioning, but they are not strict like-for-like benchmarks for the Stenutz setting. Accordingly, the contribution of the present work lies not only in predictive performance, but also in providing an early systematic machine-learning study under the substantially more difficult regime of heterogeneous experimental dipole labels.

**Table 3:** Dataset-level comparison of representative dipole-moment prediction studies

Study	Dataset	Label source	Molecular domain	Structural input	Benchmark character
Present study	Stenutz compilation	Experimental	Broad organic small molecules	3D structures not directly provided; reconstructed from name-resolved compounds	Heterogeneous experimental benchmark with substantial structural–condition ambiguity, real-measurement variability, and limited standardization relative to DFT-driven datasets.
Pereira et al. [33]	Custom DFT dataset (10,071 molecules; ZINC/GDB-13 derived)	DFT-derived	Organic molecules containing H, C, N, O, F, S, Cl, Br, P	DFT-optimized geometries and descriptors derived from those geometries	Medium-scale theoretical benchmark focused on ML prediction of DFT dipole moments from geometry-aware descriptors and partial-charge-based features.
Wayo et al. [27]	QM9	DFT-derived	Small organic molecules	Standardized QM9 molecular graphs derived from quantum-chemically optimized structures	Large-scale theoretical benchmark for graph neural networks under a relatively clean and standardized DFT labeling regime.
Ramakrishnan et al. [40]	QM9/GDB-9 data regime	DFT-derived	134k small organic molecules with up to nine heavy atoms (CHONF)	Quantum-chemically optimized equilibrium geometries and associated molecular properties	Foundational large-scale theoretical resource underlying many later dipole-prediction studies; provides highly standardized structures and properties for method development and benchmarking.
Elhalawani et al. [28]	Diatomic dataset (273 molecules)	Mixed experimental + high-level theoretical	Diatomic molecules only	Atomic-property/descriptor-level representation rather than a broad 3D organic benchmark	Narrow, low-data, domain-specific setting focused on diatomics; chemically informative but not comparable to heterogeneous experimental prediction across broad organic molecule space.

The table summarizes the underlying data regime of each study, including the source of target values, molecular scope, structural-input availability, and benchmark character. Its purpose is to contextualize comparisons at the dataset level rather than to imply strict metric comparability across fundamentally different learning settings

*Similarity structure and twin-pair dispersion across the experimental benchmark:* As summarized in Table 4, substantial label dispersion remains even among highly similar molecules, confirming that the experimental target remains noise-affected and not fully determined by local fingerprint similarity alone. Under the relaxed twin-pair criterion (Tanimoto ≥0.95), the dispersion-derived reference is limited to $R^2 = 0.862$ with $\textrm{MAE} = 0.321\,\textrm{D}$. Tightening the threshold improves the reference only partially and non-monotonically, with the strongest values observed at intermediate thresholds (Tanimoto ≥0.97: $R^{2}_{\text {upper}}=0.906$, $\textrm{MAE}_{\text {lower}}=0.270~\textrm{D}$; Tanimoto ≥0.98: $R^{2}_{\text {upper}}=0.904$, $\textrm{MAE}_{\text {lower}}=0.261~\textrm{D}$), whereas the ultra-strict Tanimoto ≥0.99 subset gives slightly weaker values ($R^{2}_{\text {upper}}=0.890$, $\textrm{MAE}_{\text {lower}}=0.276~\textrm{D}$). This likely reflects the reduced size and representativeness of the pair set at extreme thresholds, together with residual stereochemical, conformational, tautomeric, and measurement-related variability not captured by fingerprint similarity alone.

**Table 4:** Twin-pair analysis based on molecular fingerprint similarity

Tanimoto threshold	Coverage Frac	N pairs	ABS diff mean	ABS diff median	ABS diff p90	$R^2$ reference	MAE reference
0.950	0.126	228.000	0.454	0.180	1.163	0.862	0.321
0.970	0.092	156.000	0.382	0.150	0.915	0.906	0.270
0.980	0.082	135.000	0.368	0.140	0.902	0.904	0.261
0.990	0.067	114.000	0.391	0.110	1.024	0.890	0.276

Rows report results for four Tanimoto similarity thresholds used to define near-duplicate pairs. *Coverage Frac* denotes the fraction of the dataset that participates in at least one qualifying pair, and *N pairs* is the number of near-duplicate pairs identified at that threshold. *ABS diff mean*, *ABS diff median*, and *ABS diff p90* summarize the absolute dipole-moment differences (Debye) across the selected pairs. *R*$^2$ *reference* and *MAE reference* are dispersion-derived diagnostic summaries based on pairwise label variation, reported here as similarity-conditioned reference values rather than as strict physical upper or lower bounds

A second key observation is that true near-duplicates are relatively rare in this collection, as reflected by the declining participation rate (Coverage Frac drops to 0.067 at Tanimoto ≥0.99). Moreover, even among these ultra-similar pairs, the absolute dipole-moment differences remain non-negligible (e.g., $\textrm{p90}=1.024~\textrm{D}$ at ≥0.99), implying that extremely similar chemotypes can still yield appreciable label variation. Taken together–scarcity of near-duplicates, persistent dispersion among them, and non-monotonic behavior across thresholds–these results support the interpretation that the dataset is intrinsically challenging and that robust improvements must come from learning richer, non-linear structure-property relations rather than relying on nearest-neighbor memorization. In this context, the performance of the strongest learned models indicates that the proposed framework captures predictive signal well beyond what is available from local similarity alone, while still remaining necessarily constrained by the similarity-conditioned label dispersion revealed by the twin-pair analysis.

The twin-pair analysis serves as a similarity-conditioned diagnostic reference for dipole-moment prediction rather than a physically rigorous noise bound. Because dipole moment is a geometry-sensitive vector property, high 2D fingerprint similarity provides only a limited proxy for similarity in the underlying 3D electronic distribution. Accordingly, the resulting values are interpreted here as similarity-conditioned diagnostic references derived from label dispersion among highly similar 2D chemotypes, rather than as strict physical reference values.

**Fig. 1:**
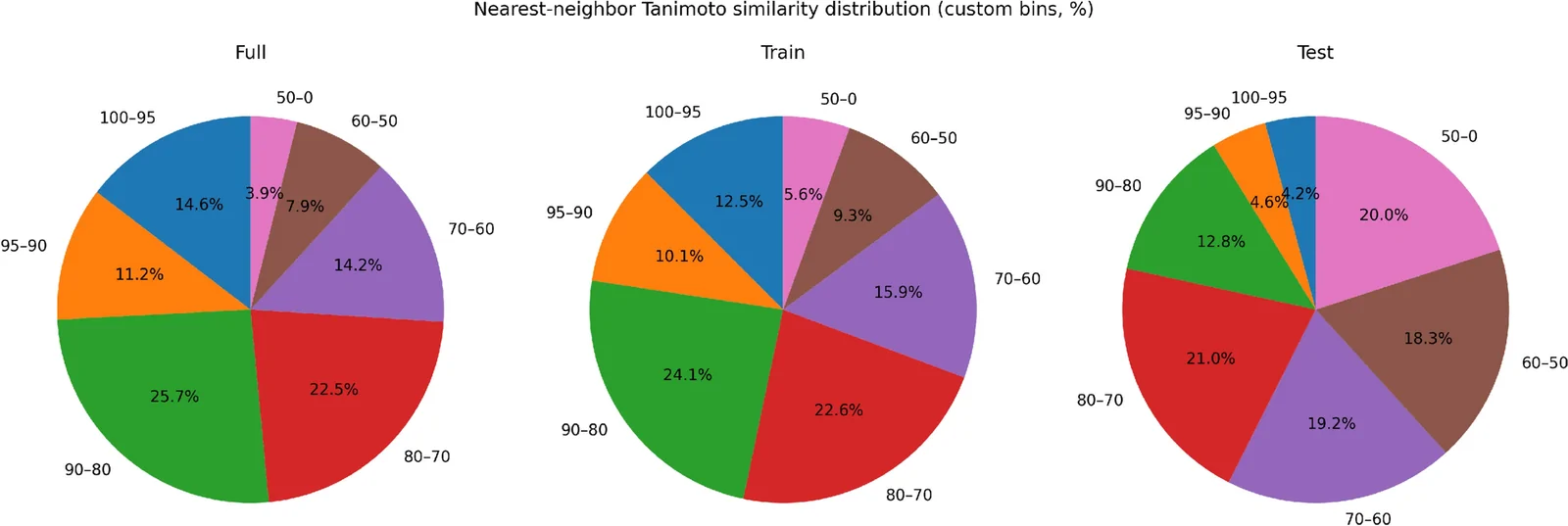
Nearest-neighbor Tanimoto similarity distribution of the experimental dataset under custom similarity bins, reported as percentages for the full collection (left), training split (middle), and held-out test split (right). For each molecule, the maximum Tanimoto similarity to its nearest neighbor (computed in the same fingerprint space used for similarity analyses) is binned into discrete intervals (e.g., 95-100%, 90-95%, …, 0-50%). The plot summarizes how frequently the benchmark contains near-duplicates versus structurally distant neighbors across splits, thereby characterizing the similarity regime in which model generalization is evaluated.

Figure 1 characterizes the intrinsic "similarity coverage" of the Stenutz.eu benchmark by reporting, for each molecule, the Tanimoto similarity to its nearest neighbor. Two observations are particularly relevant for interpreting performance under experimental noise. First, the test split contains a substantial fraction of molecules whose nearest neighbor similarity lies below the near-duplicate regime (e.g., large mass in the 50-80% bands), implying that out-of-sample evaluation frequently probes genuinely non-local generalization rather than memorization of close analogs. Second, the most stringent near-duplicate region (95-100%) constitutes only a minority of the test set, indicating that the twin-pair-derived diagnostic reference is informed by a relatively small subset and may not fully represent the full diversity encountered during testing. Together, these distributions support the view that the experimental benchmark is not dominated by trivial redundancy and that reported test performance reflects prediction under a challenging similarity regime.

#### Internal framework benchmarking and performance analysis

*Effect of multi-conformer aggregation on test-set performance:* To examine whether conformational aggregation improves predictive performance beyond a single representative geometry, we performed an additional post hoc robustness analysis using the saved full-train 3D GNN model and external test-set predictions. Three inference strategies were evaluated for the 3D branch: (i) the lowest-energy single conformer, (ii) arithmetic averaging across generated conformers, and (iii) Boltzmann-weighted averaging based on conformer energies. These 3D predictions were then combined through simple 1:1 linear fusion with the saved predictions of the extended CatBoost model in order to assess whether multi-conformer aggregation yields any additional benefit in the hybrid setting. Because this analysis depends on successful multi-conformer generation and valid 3D graph construction, the 3D GNN and hybrid results are reported on the corresponding valid subset rather than as the primary full-benchmark comparison.

As shown in Table 5, the standalone CatBoost model remained stronger than the standalone 3D GNN under all aggregation schemes, with a test-set performance of $R^2 = 0.818$, MAE =0.443, and RMSE =0.700. For the 3D GNN branch, the three inference variants produced very similar results. The single-conformer setting yielded $R^2 = 0.727$, while arithmetic averaging and Boltzmann-weighted aggregation produced $R^2 = 0.728$ and 0.727, respectively. The same pattern was observed for MAE and RMSE, indicating that multi-conformer aggregation did not substantially alter the predictive behavior of the 3D GNN on this dataset.

A more notable effect was observed in the hybrid setting. Combining CatBoost with the 3D GNN improved performance over CatBoost alone for all three variants. Within this robustness analysis, the arithmetic-mean hybrid variant gave the strongest numerical result, achieving $R^2 = 0.832$, MAE =0.391, and RMSE =0.664 on the 3D-valid test subset (n=885, Table 5). The single-conformer and Boltzmann-weighted hybrids produced nearly identical results, with only marginal numerical differences. Taken together, these findings indicate that the 3D GNN contributes complementary information to the tabular CatBoost branch, whereas explicit multi-conformer aggregation at inference time provides only limited additional benefit under the present protocol. Accordingly, this conformer-ensemble analysis is best interpreted as a robustness-oriented control rather than a full resolution of experimentally relevant conformer effects, since the remaining limitation likely reflects not only single-conformer choice but also the broader mismatch between RDKit-generated gas-phase conformer ensembles and true experimental molecular populations.

**Table 5:** Test-set performance in a post hoc multi-conformer robustness analysis

Model	$R^2$	MAE	RMSE	*n*
CatBoost	0.818	0.443	0.700	996
GNN_single	0.727	0.466	0.847	885
GNN_mean	0.728	0.467	0.846	885
GNN_boltz	0.727	0.466	0.847	885
Hybrid_single	0.832	0.391	0.665	885
Hybrid_mean	0.832	0.391	0.664	885
Hybrid_boltz	0.832	0.391	0.665	885

The CatBoost row reports full-test performance from the saved extended tabular model on all available test molecules (n=996), whereas the 3D GNN and hybrid rows are evaluated on the subset with successful multi-conformer generation and valid downstream 3D GNN outputs (n=885). Hybrid predictions were obtained by simple 1:1 linear fusion of the CatBoost predictions with the corresponding single-conformer, arithmetic-mean, or Boltzmann-weighted 3D GNN predictions. Values are rounded to three decimal places

An additional practical point emerging from Table 5 concerns sample coverage. Whereas the standalone CatBoost model produces predictions for the full external test set (n=996), all 3D GNN-based evaluations, including the hybrid variants, are restricted to n=885, reflecting the loss of 111 compounds during 3D conformer generation and graph construction (approximately 11–12% of the test set). This difference is not trivial from an application perspective. In settings where a purely 3D pipeline would return no prediction for this subset, the fusion strategy remains valuable because it preserves predictive capacity through the tabular branch while still benefiting from complementary geometric information whenever valid 3D inputs are available. In other words, hybridization is important not only for improving accuracy (e.g., CatBoost: $R^2=0.818$ vs. Hybrid_mean: $R^2=0.832$ in Table 5), but also for mitigating the practical impact of 3D-input failure by avoiding complete loss of prediction for compounds that are difficult to process in the GNN branch. Therefore, beyond its predictive advantage, the hybrid framework also offers a practical safeguard against branch-specific missingness by preserving usable predictions when one of the base models fails to produce valid outputs.

*Complexity Ladder and Low-Complexity Baseline Analysis:* The complexity-ladder analysis was designed to determine whether the predictive gains of the final framework arise primarily from increased model sophistication or from the progressive enrichment of molecular representation. The lowest-complexity baselines, based solely on direct charge-and-coordinate dipole approximations, performed poorly on the unseen test set. For example, the best direct dipole-only variant (Table 6), MMFF multi-conformer mean, reached only $R^2=-0.08$ with MAE =0.94, while several related variants remained similarly weak or even more negative. Additional conformer-robustness analyses showed that replacing single-conformer estimates with mean, median, or Boltzmann-weighted aggregation did not materially change this conclusion, with the best such variant still yielding only $R^2=-0.05$ and MAE =0.98. These results indicate that experimental dipole moments cannot be recovered adequately through direct low-level charge-based approximations alone, even when several conformers are considered.

To further test whether this weak performance was primarily driven by conformer selection, a separate conformer robustness analysis was conducted using single-conformer, arithmetic mean, median, and Boltzmann-weighted aggregation schemes for both Gasteiger- and MMFF-based dipole proxies. Across all variants, the overall conclusion remained unchanged, with all direct charge-based formulations performing substantially below the descriptor-based and hybrid learning settings. Detailed numerical results for the direct charge-based conformer robustness analysis, together with conformer-generation success statistics, are provided in the Supplementary Information

**Table 6:** Test-set summary of low-complexity baselines, descriptor-based models, and main framework components.

Group	Best model	Test $R^2$	Test MAE	Feature complexity level	Feature representation
Main model	Hybrid 2D-3D ensemble	0.84	0.40	Very high	2D + 3D hybrid
Main component	3D GNN	0.79	0.43	High	3D
Main component	CatBoost	0.82	0.45	Moderate	2D + 3D
Direct dipole calculation only	MMFF multiconf mean	− 0.08	0.94	Very low	1D
Direct dipole calculation only	MMFF single	-0.06	1.00	Very low	1D
Direct dipole calculation only	Gasteiger single	− 0.19	1.17	Very low	1D
Conformer robustness (direct dipole)	MMFF median	− 0.05	0.98	Very low	1D
Conformer robustness	MMFF single	− 0.06	1.00	Very low	1D
Conformer robustness	Gasteiger median	− 0.12	1.12	Very low	1D
3D descriptors only	Random Forest	0.21	1.07	Low	3D
3D descriptors only	ExtraTrees	0.21	1.07	Low	3D
3D descriptors only	CatBoost	0.19	1.09	Low	3D
2D + 3D descriptor fusion	CatBoost	0.74	0.58	High	2D + 3D
2D + 3D descriptor fusion	Random Forest	0.73	0.54	High	2D + 3D
2D + 3D descriptor fusion	Ridge	0.55	0.78	High	2D + 3D
Complexity ladder—only RDKit dipole features	CatBoost	0.52	0.73	Very low	1D
Complexity ladder—only RDKit dipole features	ExtraTrees	0.51	0.71	Very low	1D
Complexity ladder—only RDKit dipole features	Random Forest	0.48	0.72	Very low	1D
Complexity ladder—only 3D descriptors	ExtraTrees	0.22	1.08	Low	3D
Complexity ladder – only 3D descriptors	CatBoost	0.21	1.09	Low	3D
Complexity ladder – only 3D descriptors	Random Forest	0.21	1.08	Low	3D
Complexity ladder—only 2D fingerprints	SVR	0.75	0.53	Moderate	2D
Complexity ladder – only 2D fingerprints	CatBoost	0.73	0.58	Moderate	2D
Complexity ladder – only 2D fingerprints	Random Forest	0.70	0.56	Moderate	2D
Complexity ladder—2D + simple 3D	SVR	0.80	0.44	High	2D + 3D
Complexity ladder—2D + simple 3D	ExtraTrees	0.77	0.44	High	2D + 3D
Complexity ladder—2D + simple 3D	CatBoost	0.77	0.50	High	2D + 3D

The results show that performance improves markedly when moving from direct 1D dipole proxies or isolated 3D descriptors to combined 2D+3D representations, with the final hybrid 2D-3D ensemble providing the strongest overall performance

A similarly limited trend was observed for simple 3D descriptor models. When classical 3D shape descriptors were used in isolation, the resulting models captured only modest predictive signal, with the best held-out result reaching approximately $R^2=0.22$ and MAE =1.08 (Table 6). Such a performance remained substantially below all principal framework components and far below the final hybrid ensemble. Accordingly, while classical 3D descriptors do encode some structural information relevant to dipole behavior, they appear insufficient on their own to model the experimental target with high fidelity.

The 2D representation space remained a strong and highly competitive source of predictive signal. Under the same controlled split, the best 2D-only classical model was the fingerprint-based SVR, which reached $R^2=0.75$ with $\textrm{MAE}=0.53$ (Table 6), showing that a substantial fraction of the learnable variation is already recoverable from 2D chemical structure alone. However, the stronger CatBoost result in the main framework ($R^2=0.82$, $\textrm{MAE}=0.45$) should not be interpreted as a purely 2D outcome, since this component operates on an enriched tabular 2D+3D representation that combines fingerprints with auxiliary physicochemical, dipole-related, and conformer-dependent 3D shape descriptors. The overall pattern, therefore, suggests that 2D information provides the dominant predictive backbone, whereas 3D information becomes most useful when added in a complementary manner rather than used as an isolated representation.

The most consistent pattern in Table 6 is the performance gain associated with combining 2D and 3D information. In the classical complexity ladder, moving from 2D-only features ($R^2=0.75$, MAE =0.53) to 2D + simple 3D features increased performance to $R^2=0.80$ with MAE =0.44. A related trend was also observed in the descriptor-fusion setting, where 2D + 3D descriptor fusion reached $R^2=0.74$, clearly outperforming the 3D-only descriptor regime ($R^2\approx 0.21$). The recurrence of this improvement across multiple model classes and feature constructions indicates that the gain is not attributable to a single favorable learner, but rather to the complementary nature of 2D and 3D representations themselves.

A further methodological distinction arises from the way 3D information is introduced into the classical baselines versus the proposed framework. In the final architecture, explicit 3D information was incorporated at two levels: first within the enriched CatBoost branch through dipole-related proxy variables and conformer-dependent 3D shape descriptors, and second through the external 3D GNN branch operating on conformer-derived molecular graphs. By comparison, the "2D + simple 3D" setting in the complexity ladder combined 2D fingerprints with 3D descriptors and direct dipole-like scalar proxies, including shape descriptors such as PMI1–3, NPR1–2, radius of gyration, inertial shape factor, asphericity, eccentricity, and spherocity index, together with charge-derived dipole summaries. These classical 3D features offer several practical advantages, including ease of computation, interpretability, and direct compatibility with tabular learning algorithms. At the same time, they impose a stronger manual summarization of geometry and may discard local relational structure that can be retained in a graph-based 3D representation. The 3D descriptors, therefore, constitute a meaningful and competitive baseline, but they are not equivalent to geometry-aware message passing over molecular graphs. Their exclusion from the main model was intended to isolate the effect of the proposed architecture itself, so that any performance gain could be attributed more clearly to representation integration and model design rather than to the addition of auxiliary tabular 3D descriptors.

A related result is that direct RDKit-derived dipole proxies become substantially more useful when treated as auxiliary descriptors rather than as standalone predictors. When used in isolation, their predictive quality remained poor; however, when introduced as tabular features within the complexity ladder, the best model based only on RDKit dipole-derived features achieved $R^2=0.52$ with MAE =0.73. Although this level remains well below the best 2D-only and 2D+3D configurations, it is clearly superior to the raw direct-calculation regime. This distinction suggests that such low-level 3D-derived scalar summaries do capture useful signal, but only as part of a broader feature space rather than as a sufficient representation in their own right.

Overall, the results indicate that the strongest gains do not arise merely from increasing model complexity. Rather, they emerge from progressively enriching the representation space, especially by coupling strong 2D encodings with complementary 3D information. This interpretation is consistent with the fact that the final hybrid 2D–3D ensemble achieved the best overall test performance ($R^2=0.84$, $\textrm{MAE}=0.40$) (Table 6), exceeding both the best simple 2D+3D classical baseline ($R^2=0.80$, $\textrm{MAE}=0.44$) and each individual main component, including the 3D GNN ($R^2=0.79$, $\textrm{MAE}=0.43$) and CatBoost ($R^2=0.82$, $\textrm{MAE}=0.45$). The resulting picture is therefore more specific than a generic “higher complexity yields better performance” argument: predictive accuracy improves most consistently when complementary 2D and 3D feature spaces are integrated, and only thereafter does additional architectural sophistication provide a smaller but still meaningful final improvement.

*Internal benchmarking of the component models and ensemble variants:* A clear performance ordering is observed across the evaluated component models and linear ensemble variants. Among the single-model baselines, the fingerprint-only CatBoost model (CatBoost 1) provides the weakest overall performance, with hold-out metrics of $R^{2}=0.72\pm 0.04$, $\textrm{MAE}=0.56\pm 0.04~\textrm{D}$, and $\textrm{RMSE}=0.91\pm 0.15$, whereas the descriptor-enriched CatBoost variant (CatBoost 2) improves these values to $R^{2}=0.77\pm 0.04$, $\textrm{MAE}=0.49\pm 0.03~\textrm{D}$, and $\textrm{RMSE}=0.83\pm 0.15$. The standalone 3D GNN remains competitive, but it does not exceed CatBoost 2 at the internal hold-out level, yielding $R^{2}=0.69\pm 0.07$, $\textrm{MAE}=0.53\pm 0.04~\textrm{D}$, and $\textrm{RMSE}=0.87\pm 0.18$ (Table 7). These results indicate that strengthening the tabular branch through the extended descriptor set produces a clear gain before any explicit model-level fusion is applied.

**Table 7:** Internal benchmarking results of the component models and linear hybrid ensemble variants

Model	Hold-out			Unseen test		
	$R^{2}$	MAE	RMSE	$R^{2}$	MAE	RMSE
3D GNN	0.69 ± 0.07	0.53 ± 0.04	0.87 ± 0.18	0.74 ± 0.00	0.51 ± 0.01	0.80 ± 0.01
CatBoost 1	0.72 ± 0.04	0.56 ± 0.04	0.91 ± 0.15	0.76 ± 0.00	0.52 ± 0.01	0.77 ± 0.01
CatBoost 2	0.77 ± 0.04	0.49 ± 0.03	0.83 ± 0.15	0.79 ± 0.00	0.45 ± 0.00	0.71 ± 0.00
Ensemble 1	0.74 ± 0.06	0.49 ± 0.04	0.80 ± 0.18	0.80 ± 0.01	0.46 ± 0.01	0.71 ± 0.02
Ensemble 2	0.78 ± 0.07	0.44 ± 0.03	0.74 ± 0.18	0.82 ± 0.01	0.42 ± 0.01	0.68 ± 0.02

Values are reported as mean ± standard deviation across five folds for hold-out and unseen-test evaluations. In this cross-validation setting, each fold-specific model is trained on a reduced fraction of the available training data relative to the final full-train model; accordingly, these results are intended to summarize robustness across training realizations rather than to replace the single full-train external test benchmark. CatBoost 1 denotes the fingerprint-only tabular baseline, CatBoost 2 denotes the enriched tabular model including the extended auxiliary descriptor set, Ensemble 1 denotes the linear fusion of CatBoost 1 with the 3D GNN, and Ensemble 2 denotes the linear fusion of CatBoost 2 with the 3D GNN

The ensemble results in Table 7 further show that linear fusion systematically improves upon the individual component models, particularly when the stronger tabular branch is used. Ensemble 1, which combines CatBoost 1 with the 3D GNN, improves the unseen-test performance to $R^{2}=0.80\pm 0.01$ with $\textrm{MAE}=0.46\pm 0.01~\textrm{D}$, confirming that the fingerprint-based 2D representation and the geometry-aware 3D branch contribute complementary predictive information. However, the strongest overall performance is obtained with Ensemble 2, which combines CatBoost 2 and the 3D GNN. This model achieves the best mean hold-out metrics, with $R^{2}=0.78\pm 0.07$, $\textrm{MAE}=0.44\pm 0.03~\textrm{D}$, and $\textrm{RMSE}=0.74\pm 0.18$, and also the best unseen-test metrics, reaching $R^{2}=0.82\pm 0.01$, $\textrm{MAE}=0.42\pm 0.01~\textrm{D}$, and $\textrm{RMSE}=0.68\pm 0.02$ (Table 7).

In summary, the results in Table 7 should be interpreted in light of the cross-validation design. Because each fold-specific model is trained on a smaller effective training subset than the final full-train model, modest downward shifts relative to the final external test benchmark are expected and should not be interpreted as contradictory evidence. At the same time, the internal benchmarking results support a progressive fusion interpretation. First, performance improves when the descriptor-enriched CatBoost 2 model replaces the fingerprint-only tabular branch. Second, an additional gain is obtained when this stronger tabular predictor is linearly fused with the 3D GNN. Accordingly, the main improvement is not attributable to the 3D branch alone, but to the combination of an enhanced tabular representation with complementary geometric information, with Ensemble 2 emerging as the strongest and most stable internal configuration across both evaluation settings.

*Effect of tautomer-aware state expansion on 3D GNN performance:* As shown in Table 8, valid graph construction remained high in both settings. In the canonical configuration, usable graphs were obtained for 3974 of 3983 training molecules and 994 of 996 test molecules, and the tautomer-aware pipeline retained similarly high practical coverage. This indicates that the additional tautomer-enumeration step did not materially reduce dataset usability; thus, the observed performance differences are more plausibly attributable to the representational effects of tautomer-state expansion itself than to sample-loss artifacts.

Table 8 further shows that tautomer-aware expansion did not improve predictive performance. The canonical 3D GNN achieved $R^2 = 0.582$, $\textrm{MAE} = 0.605~\textrm{D}$, and $\textrm{RMSE} = 1.060~\textrm{D}$, whereas the tautomer-aware pooled model yielded a substantially weaker result of $R^2 = -0.114$, $\textrm{MAE} = 0.677~\textrm{D}$, and $\textrm{RMSE} = 1.730~\textrm{D}$. In other words, tautomer-aware pooling reduced $R^2$ by 0.696 and increased the prediction error by 0.072 D in MAE and 0.670 D in RMSE.

**Table 8:** Test-set performance comparison for a dedicated tautomer-sensitivity experiment conducted on the same predefined train–test split

Model	$R^2$	MAE (D)	RMSE (D)
Canonical 3D GNN	0.582	0.605	1.060
Tautomer-aware 3D GNN	-0.114	0.677	1.730

The table reports the coefficient of determination ($R^2$), mean absolute error (MAE, in Debye), and root mean squared error (RMSE, in Debye) for separately trained canonical-only and tautomer-aware 3D GNN models. In the canonical-only setting, each molecule is represented by a single parent molecular state converted into one optimized 3D graph. In the tautomer-aware setting, each molecule is represented by multiple computationally enumerated tautomeric states, each converted into a 3D graph, and the final molecular prediction is obtained by mean pooling across tautomer-state embeddings.

The tautomer-aware 3D GNN model yielded $R^2 = -0.114$, which is still scientifically informative. If canonical single-state construction were the dominant source of label misalignment in this sensitivity experiment, tautomer-aware state pooling would be expected to recover at least part of the lost signal relative to the corresponding canonical-only setting ($R^2 = 0.582$). Instead, the observed deterioration suggests that naive tautomer expansion introduces additional ambiguity when the experimentally relevant tautomer distribution is unknown. Because the reported dipole value is likely governed by a condition-dependent state ensemble, averaging across computationally enumerated tautomers may broaden the molecular representation without correspondingly improving target alignment. Accordingly, the results support the view that unconditioned tautomer expansion alone does not resolve this mismatch, whereas a more realistic treatment would likely require condition-aware state modeling and experimentally resolved molecular-state information.

A more rigorous treatment would likely require condition-aware state modeling, explicit solvent information, or experimentally grounded estimates of tautomer populations. Therefore, in the present study, the tautomer-aware experiment should be interpreted as a sensitivity analysis showing that the main limitation is not merely the omission of alternative tautomeric graphs but the broader absence of experimentally resolved state information in the compiled dataset.

### Stepwise performance gains from representation enrichment

To make the contribution of progressive representation enrichment explicit, Table 9 summarizes the test-set performance obtained at each stage of the CatBoost-centered framework, culminating in the final hybrid model. The staged design begins with a minimal Avalon-based CatBoost baseline and then progressively extends the representation through radius-expanded Avalon fingerprints, MACCS keys, basic 2D descriptors, dipole-related proxy variables, and conformer-dependent 3D shape descriptors, before finally incorporating the external 3D GNN through linear fusion. Such an analysis shows that the reported gains emerge through a systematic sequence of representation-level improvements rather than from a single end-stage model choice alone.

**Table 9:** Stepwise test-set performance progression during staged construction of the framework

Model stage	$R^2$	MAE	RMSE	Assessment
CatBoost (default) + small Avalon	0.69	0.64	0.92	Weak baseline
CatBoost (tuned) + small Avalon	0.69	0.64	0.92	Weak baseline
CatBoost (tuned) + radius-based Avalon	0.74	0.59	0.83	Improved
CatBoost (tuned) + Avalon + MACCS	0.79	0.50	0.75	Strong
CB (...) + Basic 2D descriptors	0.78	0.51	0.76	Stable/comparable
CB (...) + Dipole proxy descriptors	0.81	0.45	0.71	High
CB (...) + 3D shape descriptors	0.82	0.44	0.69	Very high
Final hybrid	0.84	0.40	0.65	Best overall

Metrics are reported on the unseen external test set. The intermediate rows summarize staged tabular-model enrichment, whereas the final row reports the full-test benchmark of the descriptor-enriched CatBoost model linearly fused with the 3D GNN (n=996). Values are rounded to two decimal places

As shown in Table 9, the main performance gains arise not from hyperparameter tuning in isolation, but from progressively enriching the molecular representation. The minimal small-Avalon CatBoost baseline remains limited ($R^2 = 0.69$, MAE =0.64, RMSE =0.92), whereas expanding the fingerprint space to radius-based Avalon improves performance to $R^2 = 0.74$ with RMSE =0.83. A further clear gain is obtained after adding MACCS keys ($R^2 = 0.79$, MAE =0.50, RMSE =0.75), indicating that broader 2D structural coverage already strengthens the tabular learner substantially. The addition of basic 2D descriptors alone leaves performance essentially unchanged ($R^2 = 0.78$, MAE =0.51, RMSE =0.76), suggesting that descriptor blocks differ in their individual informativeness when introduced in isolation. In contrast, the addition of dipole-related proxy variables produces a marked increase ($R^2 = 0.81$, MAE =0.45, RMSE =0.71), and the subsequent inclusion of conformer-dependent 3D shape descriptors raises performance again to $R^2 = 0.82$, MAE =0.44, and RMSE =0.69. Finally, the best overall result is achieved by fusing the descriptor-enriched CatBoost branch with the 3D GNN, yielding $R^2 = 0.84$, MAE =0.40, and RMSE =0.65 on the full unseen test set. This staged progression, shown in Table 9, indicates that the final performance is driven by the cumulative integration of complementary representation blocks rather than by a single end-stage model choice. In particular, the strongest gains arise as broader 2D coverage is followed by dipole-related tabular enrichment, conformer-dependent 3D descriptors, and finally explicit 3D graph-based learning.

### Error analysis across dipole-moment ranges

To examine whether predictive error varies systematically across dipole-moment magnitude, an additional stratified error analysis was performed on the unseen test set. Molecules were grouped into fixed μ ranges (0–1, 1–2, 2–3, 3–4, 4–6, and 6+ D), and bin-wise performance was computed separately for the CatBoost branch (CB), the 3D GNN branch (GNN), and the final linear 1:1 ensemble (Ens). To ensure a fair branch-level and ensemble comparison under identical evaluation support, the analysis was restricted to the matched subset of 913 unseen-test molecules for which valid predictions were available from both CatBoost and the 3D GNN. The resulting summary is shown in Table 10.

**Table 10:** Dipole-moment-range-stratified performance comparison of CatBoost, the 3D GNN, and their linear ensemble on the common evaluable unseen-test subset

Model	μ bin (D)	% of matched subset	MAE	RMSE	Bias
CB	0–1	13.5	0.496	0.760	0.420
CB	1–2	27.2	0.294	0.444	0.175
CB	2–3	26.8	0.350	0.504	0.094
CB	3–4	17.5	0.392	0.530	-0.120
CB	4–6	12.6	0.675	0.923	-0.478
CB	6+	2.4	1.547	2.207	-1.408
CB	All	100.0	0.432	0.688	0.014
GNN	0–1	13.5	0.501	0.763	0.339
GNN	1–2	27.2	0.291	0.443	0.070
GNN	2–3	26.8	0.351	0.523	-0.068
GNN	3–4	17.5	0.444	0.651	-0.144
GNN	4–6	12.6	0.684	0.966	-0.317
GNN	6+	2.4	1.663	2.331	-1.311
GNN	All	100.0	0.445	0.726	-0.050
Ens	0–1	13.5	0.467	0.702	0.379
Ens	1–2	27.2	0.244	0.387	0.123
Ens	2–3	26.8	0.302	0.445	0.013
Ens	3–4	17.5	0.357	0.492	-0.132
Ens	4–6	12.6	0.590	0.860	-0.397
Ens	6+	2.4	1.512	2.069	-1.360
Ens	All	100.0	0.384	0.631	-0.018

To ensure a fair branch-to-branch comparison, the analysis was restricted to the 913 test molecules for which valid predictions were available from both the CatBoost and 3D GNN branches. Molecules were grouped into fixed dipole-moment magnitude ranges (μ: 0–1, 1–2, 2–3, 3–4, 4–6, and 6+ D), and the table reports the number and fraction of molecules in each bin together with MAE and RMSE (Debye) for each model. The "All" row summarizes performance over the full matched subset (n=913)

Table 10 shows that the final ensemble achieves the best overall test-set accuracy, with $\textrm{MAE}=0.384$ and $\textrm{RMSE}=0.631$, improving on both CatBoost ($\textrm{MAE}=0.432$, $\textrm{RMSE}=0.688$) and the 3D GNN ($\textrm{MAE}=0.445$, $\textrm{RMSE}=0.726$). The same pattern is observed across the low-to-moderate dipole range, especially between 1–3 D, where all models perform best and the ensemble again gives the lowest errors (1–2 D: $\textrm{MAE}=0.244$; 2–3 D: $\textrm{MAE}=0.302$). This indicates that the hybrid framework is most reliable in the dipole regime that contains the largest fraction of the test set.

By contrast, Table 10 also reveals a clear degradation at higher dipole magnitude. Above 4 D, all three models show larger MAE and RMSE values together with increasingly negative bias, indicating systematic underprediction of strongly polar molecules. For the ensemble, the bias reaches -0.397 in the 4–6 D bin and -1.360 in the 6+ D bin, while MAE rises to 0.590 and 1.512, respectively. Thus, although the ensemble improves upon both individual branches across all bins, high-μ molecules remain the most challenging regime in this experimentally heterogeneous benchmark.

The bin-wise error structure in Table 10 also provides a useful explanation for the behavior of the global metrics reported above. In particular, $R^2$ is more strongly penalized by large errors on high-magnitude targets than MAE, because those deviations contribute disproportionately to the residual sum of squares. Thus, when strongly polar molecules are underpredicted—as seen from the increasingly negative bias and rising MAE/RMSE in the 4–6 D and 6+ D bins—the impact on overall $R^2$ can be substantial even if performance remains relatively strong across the majority low-to-moderate μ range. From this perspective, Table 10 further underscores that the difficulty of the dataset is not only a matter of average absolute error, but also of how a relatively small but challenging high-μ subset influences variance-based performance measures.

For this stratified error analysis, restricting evaluation to the matched 913-molecule subset was methodologically important because it allowed CatBoost, the 3D GNN, and the ensemble to be compared on exactly the same molecules. Accordingly, the metrics reported in Table 10 differ slightly from the full-test results reported elsewhere in the manuscript, where the CatBoost branch was evaluated on the complete 996-molecule unseen test set. On this matched subset, the final linear ensemble achieves $\textrm{MAE}=0.384$ and $\textrm{RMSE}=0.631$, outperforming the corresponding branch-level results on the same evaluation support and thereby providing a clearer estimate of the practical gain obtained through hybridization under directly comparable conditions. This matched-subset analysis should therefore be interpreted as a conservative but internally consistent evaluation setting, rather than as a replacement for the full-test benchmark. More broadly, the need for such matching also highlights a practical limitation of the current 3D branch: approximately 9% of the unseen test molecules and about 10% of the training molecules were not retained by the downstream 3D pipeline because valid graph-based inputs could not be constructed. As a result, the current hybrid estimates likely understate the full potential contribution of geometry-aware learning. In principle, broader and more reliable 3D coverage across both training and test partitions could further strengthen the contribution of the 3D branch and, in turn, improve the overall hybrid framework.

### Chemical diversity and benchmark noise characterization

**Fig. 2:**
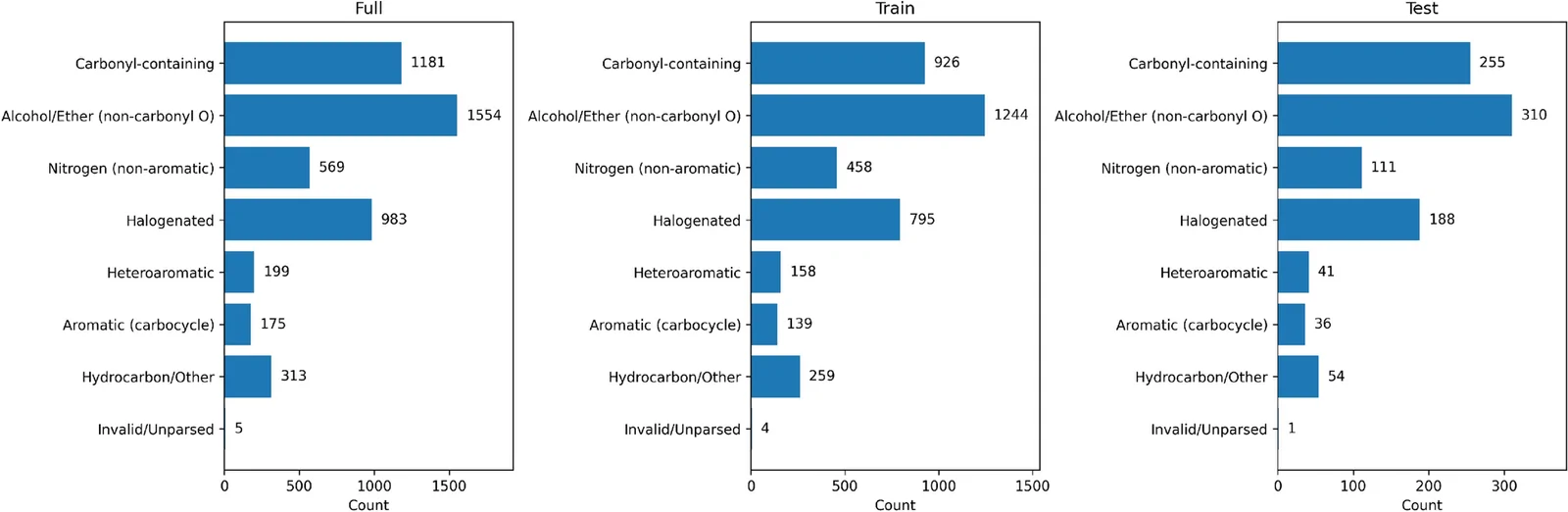
Primary chemical group distribution (main; 8-class, hierarchy-adjusted) across dataset splits. Horizontal bar charts report the number of molecules assigned to each mutually exclusive primary group–Carbonyl-containing, Alcohol/Ether (non-carbonyl O), Nitrogen (non-aromatic), Halogenated, Heteroaromatic, Aromatic (carbocycle), Hydrocarbon/Other, and Invalid/Unparsed–in the Full dataset (left), Train split (center), and Test split (right). Numeric labels at the end of each bar denote the corresponding counts.

Figure 2 summarizes the coarse-grained functional composition of the benchmark using an exclusive, hierarchy-adjusted 8-class grouping scheme. Two observations are central for interpreting the difficulty of the task. First of all, the dataset is chemically heterogeneous: the dominant presence of Alcohol/Ether (non-carbonyl O) and Carbonyl-containing molecules, together with substantial Halogenated and Nitrogen (non-aromatic) fractions, indicates a broad functional landscape rather than a narrowly focused chemical series. This matters because dipole moment is highly sensitive to both local functional-group polarity and global substitution patterns; therefore, a benchmark spanning multiple polarity-driving motifs and mixed heteroatom/halogen patterns is expected to exhibit larger intrinsic variance and a wider range of structure-property mappings. In other words, the figure supports the interpretation that the dataset is not dominated by a single chemical family and that the model must generalize across multiple functional regimes.

Figure 2 shows that the train/test distributions closely mirror the full-set composition across all eight classes, indicating that the split preserves the benchmark’s chemical breadth and avoids a trivial shift in functional-group prevalence between training and evaluation. This reduces the likelihood that performance changes arise from simple covariate shift at the level of primary chemical classes. However, the same figure also highlights why the benchmark remains noisy and challenging despite balanced splits: these classes are intentionally coarse and collapse many distinct local environments into a single label (e.g., Carbonyl-containing spans ketones, aldehydes, acids, amides, esters, and conjugated systems; Alcohol/Ether spans phenols, polyethers, and mixed heteroatom motifs). Dipole moment can vary dramatically within each class due to subtle substitutions, conformational preferences, and measurement variability; consequently, even a small change in intra-class composition (on the order of only a few percent) can translate into a disproportionate change in the apparent signal-to-noise ratio. Therefore, Fig. 2 provides supporting evidence that the benchmark is both (i) structurally diverse and (ii) inherently noisy at fine chemical resolution, making high test-set performance contingent on learning robust, transferable patterns rather than memorizing overrepresented chemotypes.

**Fig. 3:**
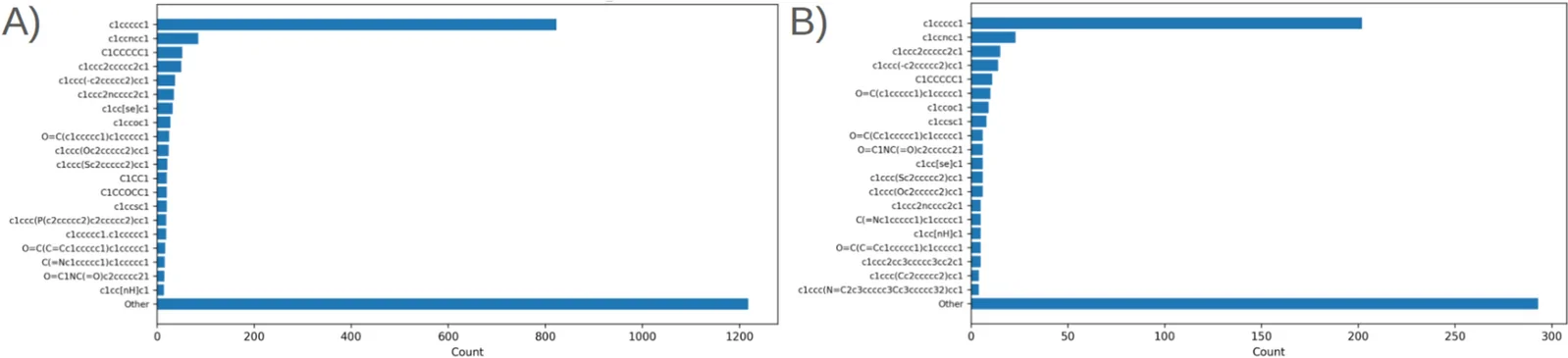
Top Bemis–Murcko scaffold frequency in the benchmark and held-out split. Horizontal bar charts show the counts of the top-20 most frequent Bemis–Murcko scaffolds (SMILES representation) with an additional “Other” category aggregating all remaining scaffolds. Panel A corresponds to the full dataset, and Panel B corresponds to the test set.

Figure 3 reports the frequency of the top Bemis-Murcko scaffolds for the full dataset (Panel A) and the unseen test set (Panel B), with all remaining scaffolds aggregated into an Other category. The most salient feature is the pronounced long-tail behaviour: although a small number of scaffolds appear repeatedly, a very large fraction of the benchmark is distributed across many low-frequency scaffolds captured by the Other bar. This pattern is a direct proxy for chemical diversity at the core-framework level, indicating that the benchmark is not dominated by a single congeneric series but rather spans numerous distinct chemotypes. For structure-property learning, such a distribution increases task difficulty because the model must generalize across scaffold families with limited within-scaffold replication, rather than exploiting dense, series-like correlations that often inflate performance in narrow chemical spaces.

The scaffold imbalance (Fig. 3) also provides an explanation for the benchmark’s effective noisiness. When most scaffolds are rare, many test compounds have weak scaffold-level support in training, and predictions rely more heavily on subtle substituent patterns and local environments rather than memorized scaffold-specific trends. In this regime, even modest experimental variability in dipole measurements can have an outsized impact on apparent model performance because the signal per scaffold is inherently undersampled. Importantly, the qualitative similarity of Panel A and Panel B suggests that the train-test split preserves the scaffold-frequency landscape (i.e., the test set is not artificially enriched with a few dominant scaffolds), which strengthens the claim that reported test performance reflects true out-of-sample generalization across a heterogeneous chemical universe. Consequently, Fig. 3 supports the interpretation that the benchmark is both scaffold-diverse and intrinsically challenging, where performance is constrained not only by model capacity but also by sparse scaffold coverage and the dataset’s experimental noise floor.

**Fig. 4:**
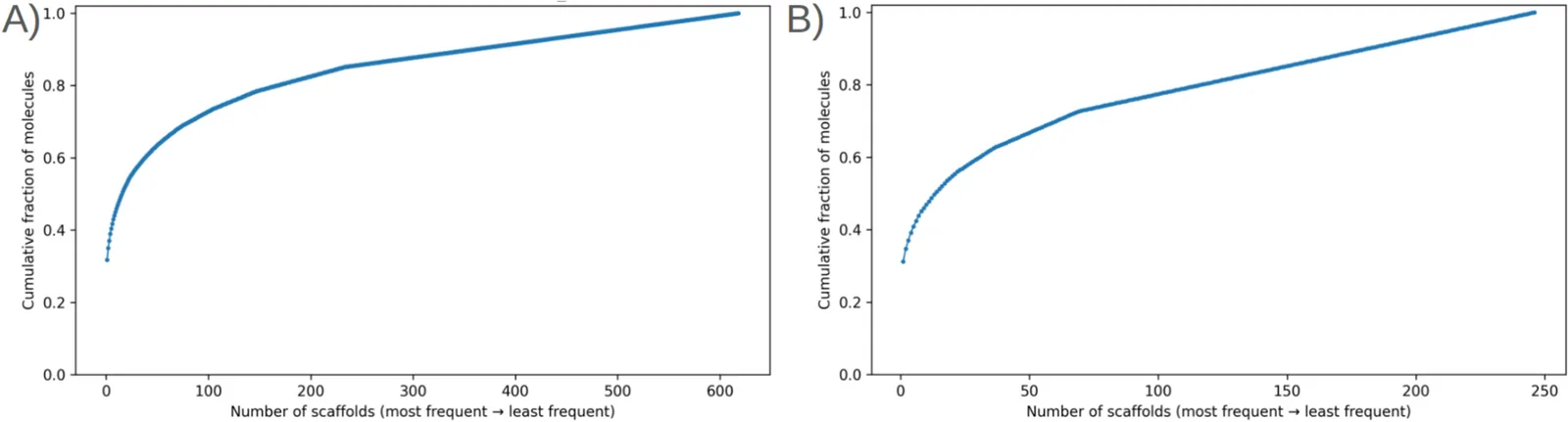
Cumulative Bemis–Murcko scaffold coverage for the full dataset and unseen test set. The curves plot the cumulative fraction of molecules (y-axis) explained as scaffolds are added in descending order of frequency (x-axis; most frequent → least frequent). Panel A corresponds to the full dataset, and Panel B corresponds to the test set.

Figure 4 quantifies scaffold diversity via cumulative Bemis-Murcko coverage as scaffolds are added from most to least frequent. The strongly concave shape in both panels indicates a classic long-tail distribution: the earliest (most frequent) scaffolds account for a substantial fraction of molecules, but achieving near-complete coverage requires incorporating hundreds of additional, progressively rarer scaffolds. Such a behaviour is a direct indicator of broad chemotype diversity at the core-framework level. Unlike series-centric benchmarks where a small set of scaffolds can explain most of the dataset, the benchmark requires many distinct scaffolds to approach full coverage, implying that a large portion of molecules belongs to sparsely populated scaffold families.

Such a long-tail scaffold structure (Fig. 4) has two important implications for perceived noise and model evaluation. First, sparse scaffold support reduces the availability of repeated, near-identical examples within each chemotype; consequently, the model must infer dipole-relevant patterns from limited within-scaffold data and transfer them across scaffolds. In such a setting, even modest experimental variability can dominate the learnable signal for rare scaffolds, amplifying the sensitivity of performance metrics to small changes in dataset composition. Second, the close qualitative agreement between the full-set (Fig. 4 Panel A) and test-set (Fig. 4 Panel B) curves suggests that the split preserves scaffold diversity rather than concentrating the test set into a few dominant frameworks. The figures strengthen the interpretation that the benchmark evaluates realistic generalization under scaffold sparsity, where performance is constrained not only by model expressivity but also by the intrinsic noise floor and the limited redundancy inherent to a chemically diverse, long-tail dataset.

Overall, this group-analysis block demonstrates that the benchmark is intrinsically challenging due to simultaneous functional heterogeneity and scaffold-level long-tail diversity. The primary-group distributions (Fig. 2) confirm that the train/test split preserves a broad chemical composition, reducing the likelihood that performance is driven by trivial covariate shift at the level of coarse functional classes. However, the same coarse grouping also implies substantial within-class variance (multiple distinct micro-environments collapsed into a single label), which naturally elevates the effective noise and limits the learnable signal in purely SMILES-derived settings. A related implication is that part of this difficulty reflects unmodeled environmental heterogeneity rather than noise in a narrow measurement-error sense alone. Since dipole moment is sensitive to phase state and solvent polarization, collapsing measurements from heterogeneous physical contexts into a single molecular target introduces context-dependent variation that is not explicitly represented in the current input space, thereby further increasing benchmark difficulty. Complementarily, the top-scaffold frequency profile (Fig. 3) and cumulative scaffold coverage (Fig. 4) reveal a pronounced long-tail chemotype distribution: a small number of scaffolds recur, but achieving broad coverage requires hundreds of low-frequency scaffolds. This sparsity reduces scaffold-level replication, increases out-of-series generalization demands, and amplifies sensitivity to experimental measurement variability. Taken together, these analyses provide a mechanistic justification for treating the benchmark as both diverse and noise-constrained, and they contextualize subsequent model comparisons as performance under a realistic, high-variance generalization regime rather than an artificially series-dominated dataset.

### Model explainability via SHAP and LIME: global and instance-level feature effects

**Fig. 5:**
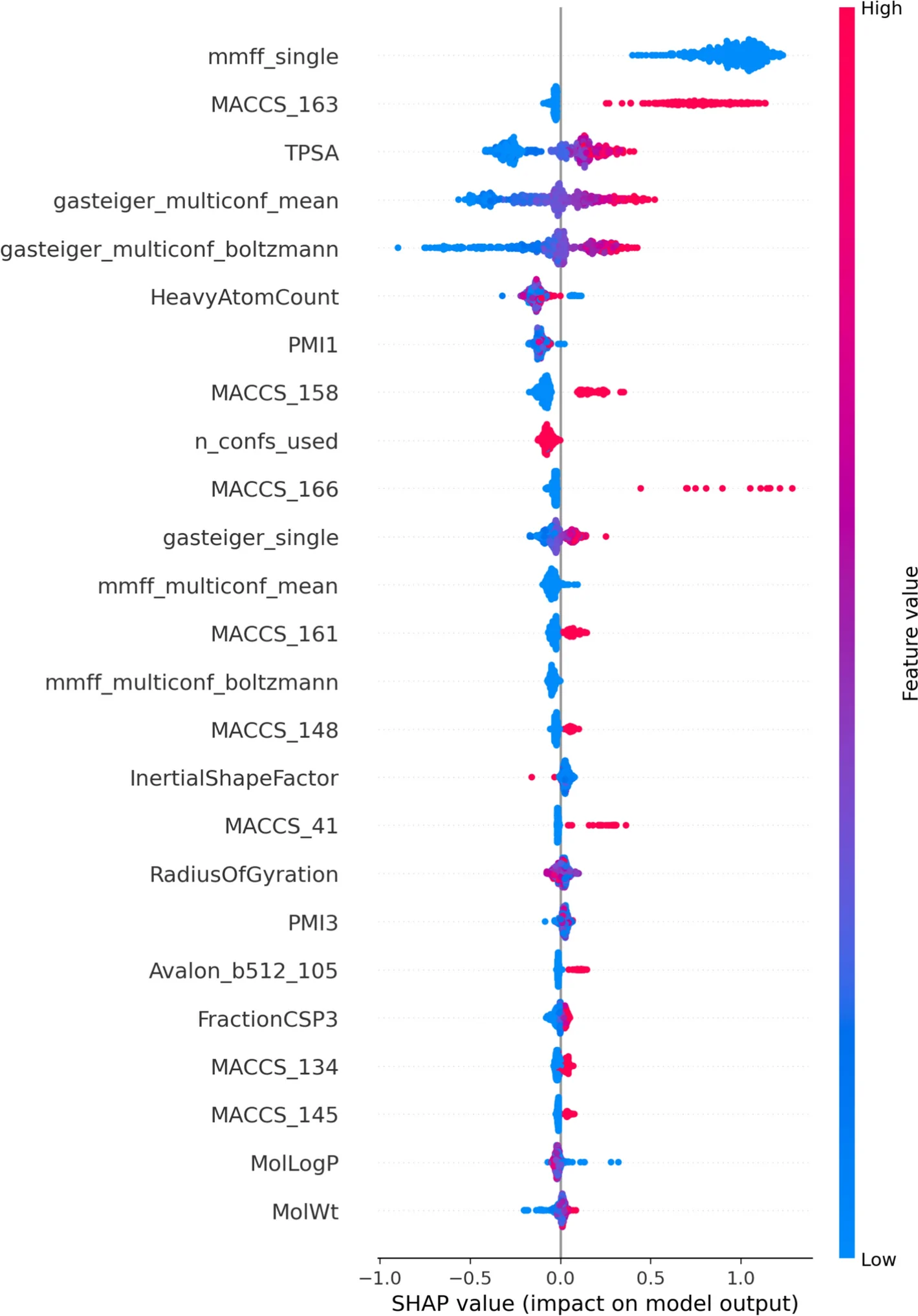
SHAP summary (beeswarm) plot of the top 25 most influential molecular input features for the trained CatBoost regressor on the test set. Features on the y-axis are ordered by mean absolute SHAP value, indicating their global contribution to model behavior. Each point represents one molecule and is positioned along the x-axis according to the SHAP value of that feature, showing whether it increases or decreases the predicted dipole moment relative to the baseline output. Point color denotes the feature value from low to high; for binary fingerprint features, this corresponds to absence/presence, whereas for continuous descriptor variables it reflects the numerical feature magnitude. To reduce overplotting, points were rendered with semi-transparency, so dense regions should be interpreted as distributional structure rather than as isolated individual points.

Figure 5 shows that the CatBoost regressor is not driven by a single feature family, but by a mixed set of dipole-related proxies, fingerprint bits, and geometry-sensitive descriptors. The strongest global contributor is mmff_single, whose high values are concentrated on the positive SHAP side, indicating that larger MMFF-derived single-conformer dipole proxies tend to increase the predicted experimental dipole moment. A similar positive trend is observed for gasteiger_multiconf_mean and gasteiger_multiconf_boltzmann, although with broader dispersion, suggesting that charge-derived multi-conformer summaries provide useful but context-dependent signal. Among continuous physicochemical descriptors, TPSA is also highly ranked and shows a predominantly positive shift at higher values, consistent with the contribution of polarity-related molecular environments to larger predicted μ.

The binary fingerprint variables likewise show clear directional behavior. In particular, the presence state of MACCS_163, MACCS_158, MACCS_166, and MACCS_161 is concentrated mainly on the positive SHAP side, indicating that the corresponding structural patterns are repeatedly associated with upward shifts in the model output. In contrast, several geometric descriptors such as PMI1, PMI3, RadiusOfGyration, and InertialShapeFactor, have narrower SHAP ranges centered closer to zero, implying that they contribute more modestly on average and likely act as secondary refinements rather than primary drivers. The feature n_confs_used is also tightly concentrated near zero, suggesting that the number of retained conformers has limited direct marginal influence relative to chemically interpretable descriptor content.

An important observation from Fig. 5 is that several features, especially TPSA, gasteiger_multiconf_mean, and gasteiger_single, exhibit points on both sides of zero across their value range. This indicates that their effect is not purely monotonic, but depends on molecular context and interaction with other variables learned by the boosted-tree ensemble. Thus, the SHAP profile supports two conclusions. First, the enriched CatBoost branch is using genuinely heterogeneous information rather than relying only on sparse 2D fingerprints. Second, the strongest explanatory variables are dominated by dipole-proxy and polarity-related terms, while selected fingerprint bits provide discrete structural corrections and shape descriptors contribute finer geometry-sensitive adjustment. Overall, the figure supports the design logic of the tabular branch: predictive signal is strongest when structural, physicochemical, and conformer-derived information are integrated within the same model.

The SHAP ranking (Fig. 5) also indicates that predictive behaviour is not governed by a single variable type, but instead emerges from the combined contribution of structural keys, physicochemical descriptors, dipole-related proxies, and conformer-sensitive geometric terms. This is informative for the modeling setup, as it confirms that the enriched CatBoost branch is utilizing a genuinely heterogeneous representation rather than relying exclusively on sparse fingerprint signals. In that sense, the SHAP analysis provides direct support for the design choice of extending the tabular branch beyond fingerprint-only inputs.

Table 11 shows that the LIME explanations are dominated by a mixture of structural fingerprint signals and descriptor-based variables rather than by a single feature family. The largest mean absolute LIME weights are observed for MACCS_163 (0.61267), MACCS_166 (0.54223), and MACCS_16 (0.53441), indicating that several binary structural keys repeatedly exert strong local influence on the model predictions. At the same time, the presence of gasteiger_multiconf_mean with a weight of 0.42538 shows that continuous charge-related descriptors also contribute substantially at the instance level.

**Table 11:** Top features identified by the LIME analysis for the Catboost model (2D+3D), ranked by mean absolute LIME weight

Feature	Mean absolute LIME weight
MACCS_163	0.61267
MACCS_166	0.54223
MACCS_16	0.53441
gasteiger_multiconf_mean	0.42538
MACCS_1	0.37629
gasteiger_multiconf_boltzmann	0.27864
Avalon_b1024_637	0.26255
Avalon_b1024_63	0.26255
MACCS_4	0.26188
MACCS_41	0.26188
MACCS_158	0.25254
MACCS_15	0.25254
Avalon_b512_179	0.23148
TPSA	0.22473
MACCS_30	0.22279

Higher values indicate a stronger average local contribution to the model predictions across the explained samples

A similar mixed pattern is retained across the rest of the ranking in Table 11. For example, gasteiger_multiconf_boltzmann reaches 0.27864, Avalon_b1024_637 and Avalon_b1024_63 both reach 0.26255, and TPSA remains among the top locally influential variables at 0.22473. Overall, these results suggest that the Catboost model (2D+3D) assembles its local predictions from both discrete substructure indicators and continuous physicochemical or charge-related descriptors, consistent with the enriched tabular design adopted in the framework.

**Fig. 6:**
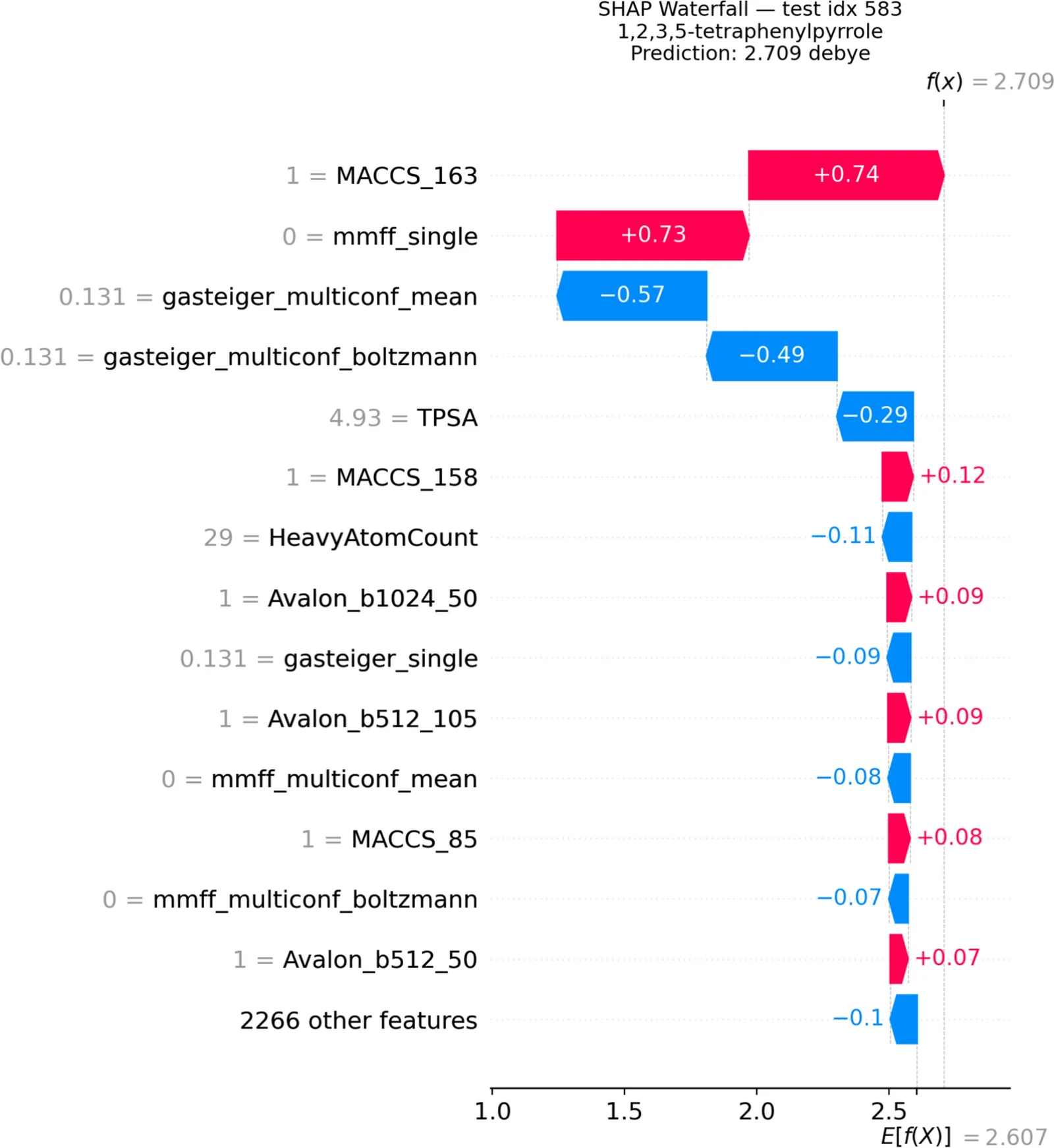
SHAP waterfall plot for the test-set molecule 1,2,3,5-tetraphenylpyrrole (test index 583). The plot decomposes the CatBoost prediction into additive feature-level contributions relative to the model baseline output. Positive SHAP values increase the predicted dipole moment, whereas negative SHAP values decrease it. The displayed bars correspond to the most influential local features for this sample, while the remaining contributions are grouped under "other features", providing a compact view of the dominant drivers of the prediction.

Figure 6 provides a local explanation for the CatBoost prediction obtained for 1,2,3,5-tetraphenylpyrrole. For this molecule, the predicted dipole moment is 2.709 debye, whereas the experimentally observed value is 2.04 debye. The waterfall decomposition shows that the final prediction is determined by the balance between several strong positive and negative feature contributions rather than by a single dominant variable. Among the largest upward contributions are MACCS_163 (+0.74) and mmff_single (+0.73), both of which shift the prediction substantially above the baseline expectation. In contrast, gasteiger_multiconf_mean (-0.57), gasteiger_multiconf_boltzmann (-0.49), and TPSA (-0.29) exert pronounced downward effects, partially counteracting the positive structural contributions. Additional smaller positive effects arise from MACCS_158 (+0.12), Avalon_b1024_50 (+0.09), Avalon_b512_105 (+0.09), MACCS_85 (+0.08), and Avalon_b512_50 (+0.07), whereas HeavyAtomCount (-0.11), gasteiger_single (-0.09), mmff_multiconf_mean (-0.08), and mmff_multiconf_boltzmann (-0.07) further moderate the prediction. Taken together, this instance-level explanation indicates that the local decision process for this molecule emerges from an interplay between binary fingerprint activations and continuous descriptor-based variables related to charge distribution, polarity, and force-field-derived geometry. From an interpretability perspective, this is important because it shows that the CatBoost model does not rely exclusively on one representation family but instead constructs its prediction through a heterogeneous set of chemically meaningful signals whose opposing effects can be traced explicitly at the single-molecule level.

**Fig. 7:**
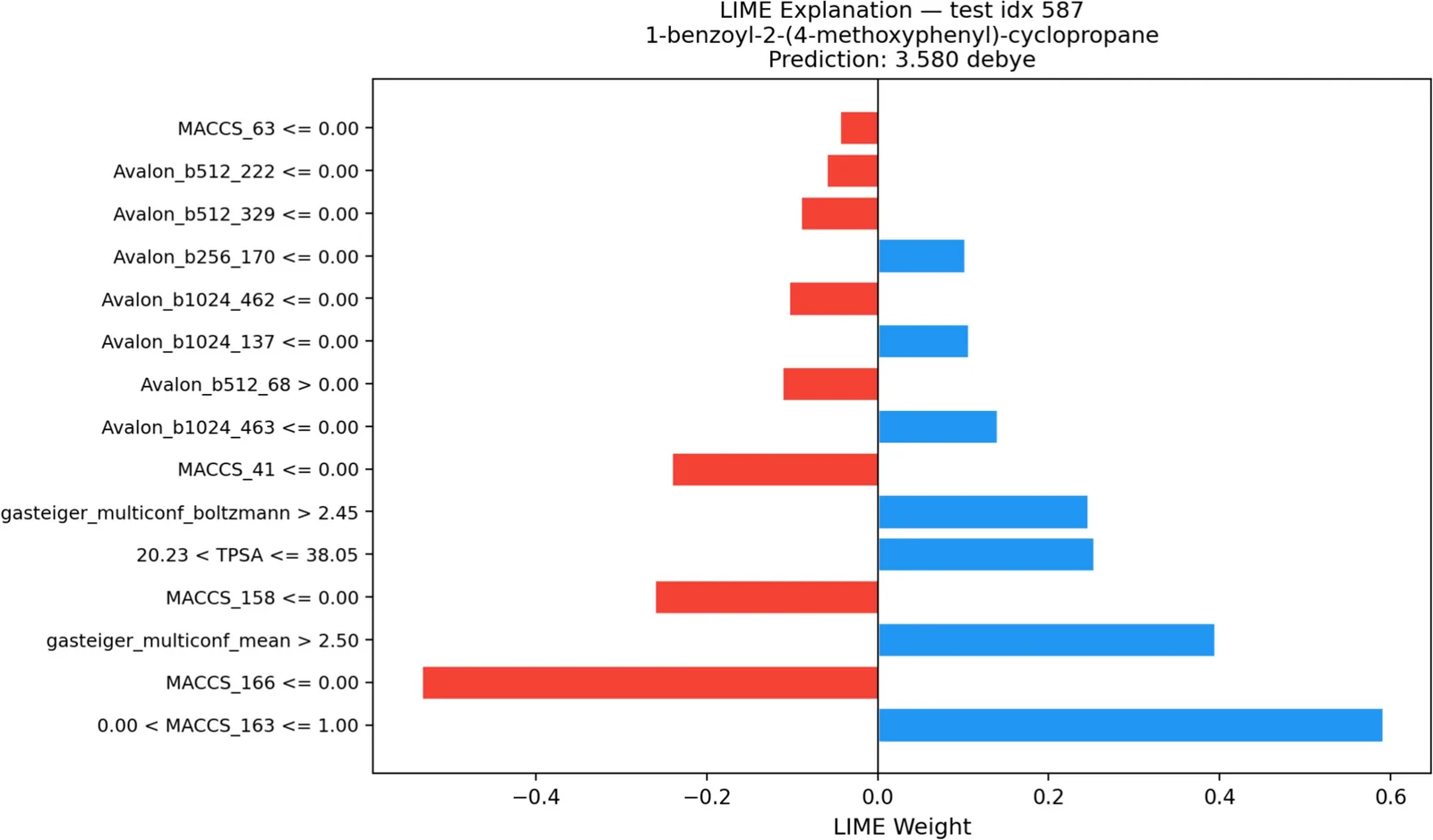
LIME explanation plot for the test-set molecule 1-benzoyl-2-(4-methoxyphenyl)-cyclopropane (test index 587). The plot shows the local feature contributions used to explain the CatBoost prediction for this sample. Bars extending to the right indicate features that increase the predicted dipole moment, whereas bars extending to the left indicate features that decrease it. The displayed conditions correspond to the most influential local decision rules identified by LIME, providing a compact local explanation of the model output for this molecule.

Figure 7 provides a local surrogate-based explanation for the CatBoost prediction obtained for 1-benzoyl-2-(4-methoxyphenyl)-cyclopropane. For this molecule, the predicted dipole moment is 3.580 debye, whereas the experimentally observed value is 2.97 debye. The LIME decomposition indicates that the local prediction is shaped by a balance between several strong positive and negative rule-based contributions. The most influential positive term is the condition $0.00 < \mathrm {MACCS\_163} \le 1.00$, with an approximate contribution of +0.59, followed by $gasteiger\_multiconf\_mean > 2.50$ (+0.40), $20.23 < \textrm{TPSA} \le 38.05$ (+0.25), and $gasteiger\_multiconf\_boltzmann > 2.45$ (+0.24). These upward-driving components are partially offset by several negative rules, most notably $\mathrm {MACCS\_166} \le 0.00$ (-0.53), $\mathrm {MACCS\_158} \le 0.00$ (-0.26), and $\mathrm {MACCS\_41} \le 0.00$ (-0.24), together with smaller negative contributions from the absence or thresholding of several Avalon features such as $\mathrm {Avalon\_b1024\_462} \le 0.00$, $\mathrm {Avalon\_b512\_68} > 0.00$, $\mathrm {Avalon\_b512\_329} \le 0.00$, and $\mathrm {Avalon\_b512\_222} \le 0.00$. Overall, this explanation suggests that the local prediction for this molecule is assembled from a heterogeneous combination of binary structural indicators and continuous descriptor-based thresholds rather than from a single dominant signal. From an interpretability perspective, this is informative because it shows that the model’s local behavior can be expressed as an additive compromise between activating and suppressing molecular conditions, thereby making the prediction pathway more transparent at the single-molecule level.

### Case study: an application of the framework

Table 12 in Supplementary Information presents molecule-level predictions for a fixed random illustrative subset of 15 compounds spanning a broad dipole-moment range. The table is not intended to highlight best-case or worst-case examples, but rather to show how the framework behaves across chemically diverse instances under the same evaluation setting.

At the single-molecule level, predictive accuracy varies across the three models, indicating that no single representation is uniformly optimal across chemical space. In several compounds, CatBoost provides the closest estimate to experiment, including (E)-2-chlorobenzaldehyde oxime (1.5154 vs. 1.63), (E)-but-2-enoic acid (2.2600 vs. 2.13), (E,E)-1-phenyl-5-(2,4,6-trimethoxyphenyl)-1,3-pentanediene-5-one (4.6179 vs. 4.59), and 9-anthrylidene-2,4,6-trimethylaniline (1.8379 vs. 1.76) (Table 12 in Supplementary Information). In contrast, the 3D GNN is more accurate for molecules such as cis-3-methylcyclohexanol (1.7041 vs. 1.91), trans-3,3’-dichlorostilbene (1.7995 vs. 1.94), (E)-1-(4-bromophenyl)-3-(1H-pyrrol-2-yl)prop-2-en-1-one (2.6407 vs. 2.52), and 2-ethoxy-1,3,2-dioxaphosphorinane (2.9413 vs. 3.05) (Table 12 in Supplementary Information). Such a compound-specific variation supports the broader interpretation that experimental dipole moments are controlled by multiple interacting determinants, including substructure composition, physicochemical context, and geometry-dependent charge arrangement.

The hybrid model is most informative when the two component learners make partially complementary errors. In some rows it is the most accurate predictor, such as (2E,4E)-1-furan-2-yl-5-phenylpenta-2,4-dien-1-one, where the hybrid estimate (3.2337) is closer to the experimental value (3.27) than either CatBoost (3.3153) or the 3D GNN (3.1520) (Table 12 in Supplementary Information). A similar pattern appears for 2-[(4-nitrophenyl)methylidene]propanedinitrile, where the hybrid prediction (3.3043) improves substantially over CatBoost (4.2168) and also remains closer to experiment than the 3D GNN (2.3919) (Table 12 in Supplementary Information). Even when it is not the row-wise best model, the hybrid frequently occupies an intermediate position between the two component predictions, consistent with partial error compensation through linear fusion.

The table also reveals chemically informative failure patterns. Several high-dipole compounds are underestimated by both component models, including (1R,4S,5R,6S)-5,6-dicyanobicyclo[2,2,2]oct-2-ene (4.3109 and 4.0668 vs. 6.53) and 3,7,10-trimethyl-2,8,9-trioxa-5-aza-1-borabicyclo[3.3.3]undecane (4.4036 and 3.5268 vs. 6.61) (Table 12). This suggests that, for some strongly polar systems, the available representations still fail to recover the full magnitude of the experimental response. In contrast, certain mid-range molecules show strong disagreement between the component models, as in 2-[(4-nitrophenyl)methylidene]propanedinitrile, where CatBoost overshoots while the 3D GNN undershoots (Table 12 in Supplementary Information). Such disagreement is itself informative because it indicates regions of chemical space in which prediction is more uncertainty-prone and model assumptions diverge.

A further observation is that the relative advantage of the two learners appears to be structure-dependent rather than governed by a simple low-μ versus high-μ separation. CatBoost performs well in several cases where enriched tabular descriptors appear sufficient to capture the dominant polarity signal, such as (E)-2-chlorobenzaldehyde oxime, (E)-but-2-enoic acid, and 9-anthrylidene-2,4,6-trimethylaniline. In contrast, the 3D GNN becomes more advantageous in compounds where spatial arrangement is likely to play a stronger role, including cis-3-methylcyclohexanol, trans-3,3’-dichlorostilbene, and 2-ethoxy-1,3,2-dioxaphosphorinane (Table 12 in Supplementary Information). This pattern is chemically plausible: the tabular branch captures stable structural and physicochemical priors, while the graph-based branch is better positioned to model geometry-sensitive vector addition and cancellation effects. Overall, Table 12 reinforces the central rationale of the framework: predictive performance improves most consistently when tabular and geometry-aware representations are used in a complementary manner. The molecule-level patterns further indicate that residual error is chemically structured rather than purely random, supporting the use of hybrid modeling for heterogeneous experimental dipole-moment prediction.

### Limitations and future work

A limitation of the present benchmark is the incomplete and inconsistently specified treatment of stereochemistry. As shown in Table 13 in Supplementary Information, molecules for which stereochemistry could plausibly affect dipole behavior do not constitute a dominant fraction of the dataset: only 409 molecules (8.21%) fall into the present stereo-relevant subset, defined here as molecules capable of diastereomerism or containing at least one potential E/Z stereobond. Moreover, only 221 of these 409 molecules (54.03%) carry fully specified stereochemical annotation, implying that the remaining 188 molecules (45.97%) belong to the stereo-relevant subset but lack complete stereochemical specification. In practice, this means that for a substantial fraction of molecules whose dipole behavior could plausibly depend on diastereomerism or E/Z isomerism, the available inputs do not fully resolve the relevant structural state. Under these conditions, part of the residual error may reflect unresolved stereochemical ambiguity rather than a failure of the model to learn a well-specified stereochemical signal. Under these conditions, the current dataset does not provide a suitable basis for a systematic evaluation of stereochemistry-sensitive learning. Accordingly, stereochemistry should be regarded here primarily as a residual source of structural ambiguity rather than as a feature dimension meaningfully encoded and learned by the present models. For a geometry-sensitive property such as dipole moment, this remains a potential source of ambiguity, particularly for molecules capable of diastereomerism or E/Z isomerism, where distinct structural states may plausibly contribute different experimental μ values. A more appropriate future direction would therefore be to evaluate stereo-aware modeling only within explicitly stereo-relevant and fully specified subsets, rather than treating stereochemistry as a uniformly exploitable signal across the benchmark.

A second limitation arises from unmodeled environmental heterogeneity in the experimental labels. The compiled dipole-moment values were measured under diverse physical conditions, including different solvent environments, phase states, and experimental protocols, whereas the current framework is restricted to molecule-centered structural and descriptor-based representations. As a result, residual prediction error cannot be attributed solely to measurement noise, because part of the unexplained variance likely reflects context-dependent effects that are not observed by the model. Although the expanded tabular descriptor set may capture some intrinsic tendencies related to polarity, charge distribution, and geometry, it does not provide an explicit representation of environment-specific polarization or measurement conditions. Future progress on this problem will therefore likely require either condition-aware modeling strategies or evaluation on more homogeneous experimental subsets.

A third limitation concerns the simplified treatment of conformational variability in the geometry-aware branch. Although conformational sampling was performed prior to representation construction, the 3D GNN ultimately relies on a single lowest-energy proxy geometry, and the tabular branch incorporates conformer information only through derived summary descriptors. Consequently, the framework can capture part of the geometry-sensitive signal relevant to dipole prediction, but it does not fully reflect Boltzmann-weighted conformer populations or experimentally relevant state mixtures, especially for flexible molecules. This limitation likely contributes to residual error in the experimental setting and may help explain why the hybrid model remains below the twin-pair-derived diagnostic reference. Future work should therefore explore multi-conformer inference, ensemble-level molecular representations, and calibrated uncertainty estimation strategies, including prediction intervals or conformal approaches, to better reflect both structural variability and heterogeneous experimental uncertainty.

## Conclusion

The study presents a progressive 2D-3D hybrid framework for experimental dipole-moment prediction under heterogeneous real-world labels. Rather than targeting near-noise-free theoretical benchmarks, the framework was developed for the substantially more challenging Stenutz experimental compilation, where measurement variability, stereochemical ambiguity, conformer mismatch, and incomplete structural standardization compress the practically attainable performance range. Within this setting, the modeling strategy proceeded in stages: from a fingerprint-based CatBoost baseline, to an enriched tabular model including physicochemical, dipole-related, and conformer-dependent 3D shape descriptors, and finally to linear fusion with a geometry-aware 3D GNN.

A central outcome is that performance in this regime should be interpreted relative to experimentally grounded internal anchors rather than absolute metrics alone. In the dataset, a fingerprint-based *k*-NN baseline provides a stringent lower reference ($R^2=0.65$, $\textrm{MAE}=0.64~\textrm{D}$), while twin-pair dispersion analysis defines a similarity-conditioned diagnostic reference range of approximately $R^2 \approx 0.86$–0.91 with $\textrm{MAE} \approx 0.26$–$0.32~\textrm{D}$. These two anchors provide a more realistic context for judging model performance on heterogeneous experimental dipole labels.

Within this experimentally constrained setting, the final linear hybrid achieved the best overall performance, reaching $R^2=0.844$ with $\textrm{MAE}=0.399~\textrm{D}$. This substantially exceeds the similarity baseline, remains close to the twin-pair-derived diagnostic range, and supports competitive positioning relative to prior dipole-prediction studies when differences in label regime are taken into account. However, the results also show that the main gain does not arise simply from increasing model complexity. Instead, improvement is obtained most consistently through staged representation enrichment and the combination of complementary 2D and 3D information. The enriched tabular branch already improves over the 2D baseline, and its subsequent fusion with the 3D GNN yields the strongest generalization.

## Supplementary Information

Supplementary file 1.

## Data Availability

Raw data can be found at: https://www.stenutz.eu/chem/dipole.php The data and models at: https://github.com/yauz3/dipole_moment
