# Supplementary material for: Experimental dipole moment prediction with a progressive 2D–3D hybrid framework and twin-pair-based diagnostic evaluation
Source: J Cheminform. 2026 Jun 14;18:119. doi: 10.1186/s13321-026-01215-4 (PMC13495258; doi:10.1186/s13321-026-01215-4)
Supplement: Supplementary file 1 [file 13321_2026_1215_MOESM1_ESM.pdf]

5 Supplementary Information

The supplementary information provides extended analyses and additional results that support the main conclusions while improving transparency and reproducibility. In particular, Extended Comparative Analysis Across Diverse Regression Families reports a broad, like-for-like benchmarking suite across diverse regression families under the same data split and validation protocol, with the primary aim of reducing model-selection bias and demonstrating that the observed performance trends are not artefacts of a narrow set of chosen baselines. Additional supplementary sections document implementation details, extended ablations, and auxiliary diagnostic/visualization outputs that are impractical to include in the main text but are essential for interpreting robustness under experimental noise and for enabling faithful replication of the full pipeline.

5.1 Component Analysis of the Modeling Framework

Aggregate performance metrics alone do not fully explain why a model generalizes or which representation components drive predictive gains under experimental variability. Accordingly, this subsection presents nine complementary analyses designed to characterize the data structure and to diagnose the mechanisms underlying model behavior. The analyses span (i) global trend inspection of the structure-property landscape (PCA), (ii) local manifold geometry and property smoothness (t-SNE), (iii) univariate feature relevance profiling (ANOVA F-statistics). Collectively, these views provide an integrated interpretation of representation complementarity, feature organization, and the mapping from molecular inputs to dipole-moment predictions.

5.1.1 Global Structure-Property Landscape: PCA-Based Trend Analysis

To obtain a global, low-dimensional view of the hybrid representation, principal component analysis (PCA) was applied to the feature space of the CatBoost model (2D+3D), which combines fingerprint-based inputs with the additional auxiliary 3D shape and dipole-related descriptor block. Figure 8 shows the two-dimensional PCA projection colored by the experimental dipole moment (μ, Debye), while Figure 9 summarizes the dominant feature contributions to the first two principal components. Together, these plots provide complementary information: the score plot describes how molecules are distributed in the reduced space, whereas the loading plot indicates which feature groups are most responsible for the observed global variation.

Figure 8 shows that the first two principal components explain a modest fraction of the total variance (PC1 = 7.98%, PC2 = 3.21%), indicating that the structure of the full feature space is distributed across many dimensions rather than concentrated in a single dominant linear axis. This behavior is expected for a heterogeneous hybrid representation composed of multiple substructure indicators and descriptor families. The projection reveals several visually distinct regions rather than a single continuous cloud, suggesting that the dataset contains multiple chemotype-dependent submanifolds. At the same time, the dipole-moment color gradient remains only partially organized in the PC1–PC2 plane: high-μ compounds appear as sparse pockets and isolated points rather than a compact cluster or a smooth global trajectory. This implies that dipole moment is not governed by a simple linear trend in the leading PCA directions. Instead, the target seems to depend on more localized, non-linear, or higher-order combinations of representation components. The mixed color intensities observed even within dense neighborhoods further suggest that nearby points in the global PCA projection can still differ meaningfully in μ, which is consistent with the difficulty of predicting a vector-sensitive physicochemical property from compressed 2D/descriptor-based structure summaries alone.

Figure 9 helps interpret which feature groups dominate the major axes of global variation. For both PC1 and PC2, the strongest loadings are largely assigned to fingerprint variables, with repeated contributions from both Avalon and MACCS bits. In particular, several MACCS features are among the top contributors in PC1, while PC2 is also shaped by a mixture of Avalon and MACCS components. Based on this

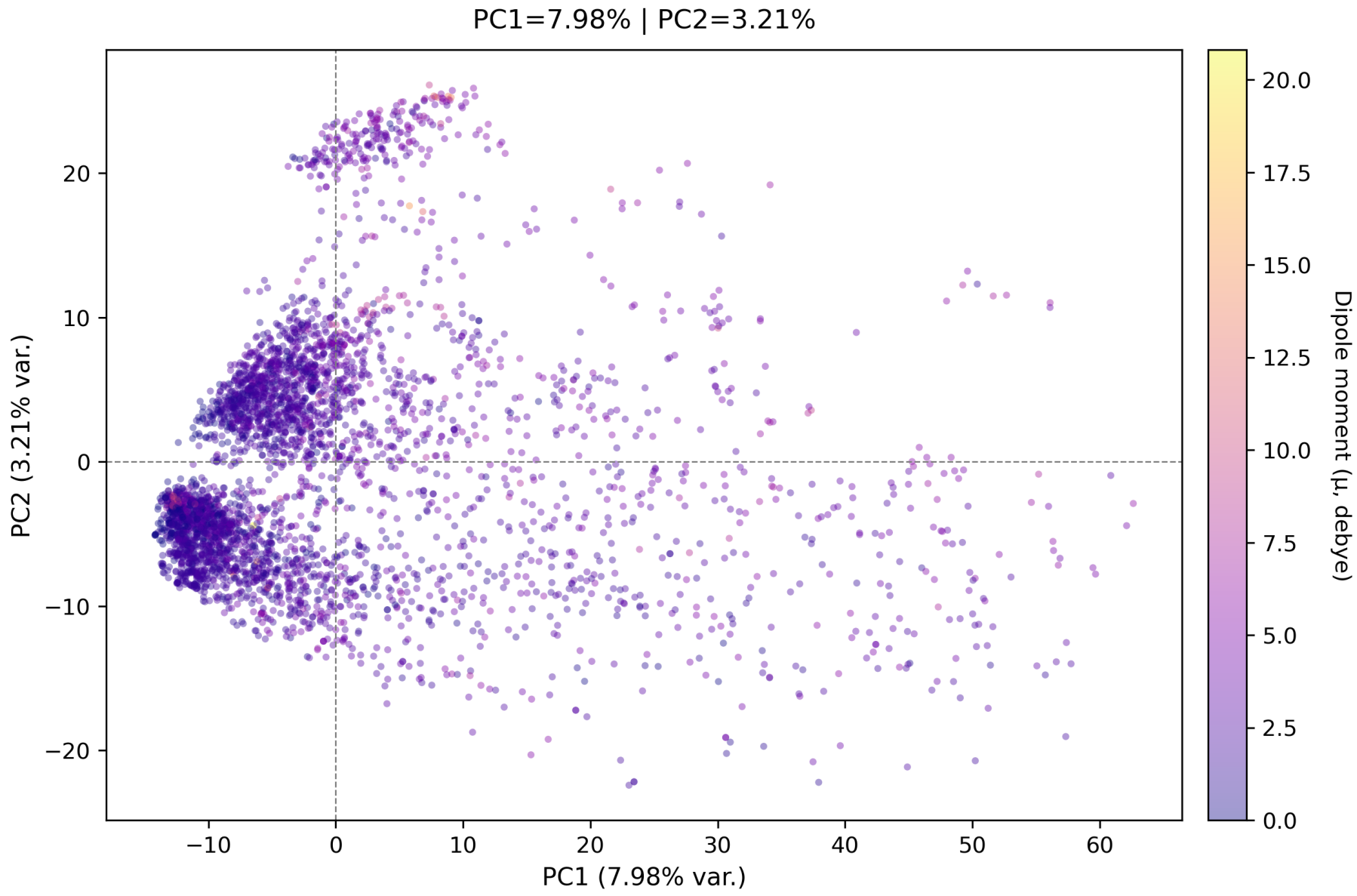

Fig. 8: Two-dimensional PCA projection of the feature space, with points colored by the experimental dipole moment (μ, Debye). The first two principal components explain 7.98% (PC1) and 3.21% (PC2) of the total variance, respectively. Each point represents one molecule in the reduced hybrid feature space. To improve readability in dense regions, points were rendered with semi-transparency, so local clusters should be interpreted as distributional patterns rather than exact point counts.

loading pattern, the most prominent source of variation in the first two principal directions appears to come from fingerprint-derived structural encoding rather than from the auxiliary continuous descriptor groups. Although this does not prove that MACCS is globally the best predictive representation, it does suggest that MACCS bits are especially influential in organizing the low-dimensional PCA landscape, and therefore may be a promising representation component in the benchmark. Nevertheless, this interpretation should remain cautious: PCA loadings reflect variance contribution to unsupervised axes, not direct target importance. Therefore, the loading profile is better viewed as evidence of representation dominance in the global geometry of the dataset, while supervised analyses such as SHAP, ANOVA, and model-specific attribution remain necessary to determine which feature groups most directly drive dipole-moment prediction.

Overall, the PCA results support two main conclusions. First, the hybrid 2D-3D space captures substantial structural diversity, but the relevant information is spread across many dimensions, limiting the interpretability of any two-component linear projection. Second, the dominant global organization of this space is driven primarily by fingerprint features—especially MACCS and Avalon bits—whereas dipole moment itself remains only partially aligned with the leading unsupervised axes. This combination of broad structural clustering and weak global property linearity provides

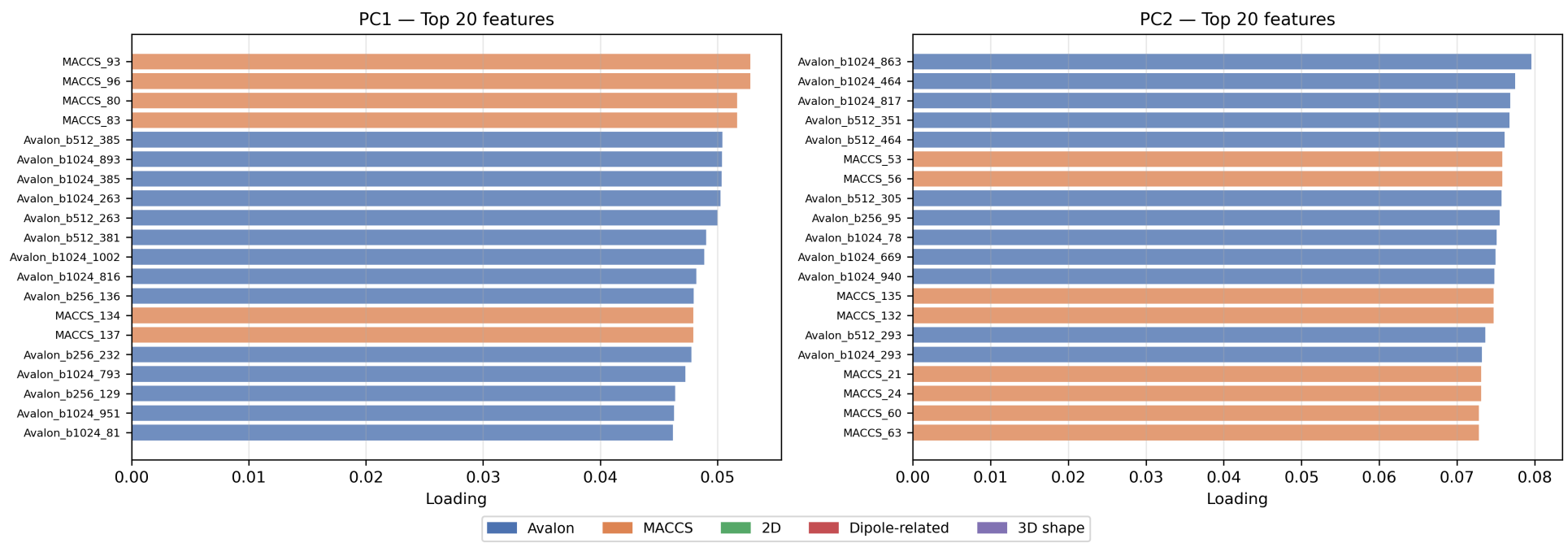

Fig. 9: Top absolute PCA loadings for PC1 and PC2 in the representation. Colors denote feature groups, including Avalon fingerprints, MACCS fingerprints, 2D descriptors, dipole-related descriptors, and 3D shape descriptors. The dominant loadings for the first two principal components are primarily associated with fingerprint-derived variables, especially Avalon and MACCS features, indicating that these 2D representations contribute most strongly to the leading axes of variance in the hybrid feature space.

further justification for flexible non-linear learners and motivates the subsequent local-manifold and feature-attribution analyses, which are better suited to resolve region-specific structure–property behavior.

5.1.2 Local Neighborhood Geometry and Property Smoothness: t-SNE Manifold Analysis

To examine the local organization of the CatBoost representation beyond global linear structure, t-SNE was applied to the feature space corresponding to the extended descriptor set used in the final tabular branch. Figure 10 shows the resulting two-dimensional manifold colored by the experimental dipole moment (μ, Debye), allowing qualitative inspection of local neighborhood structure and property smoothness in the enriched representation.

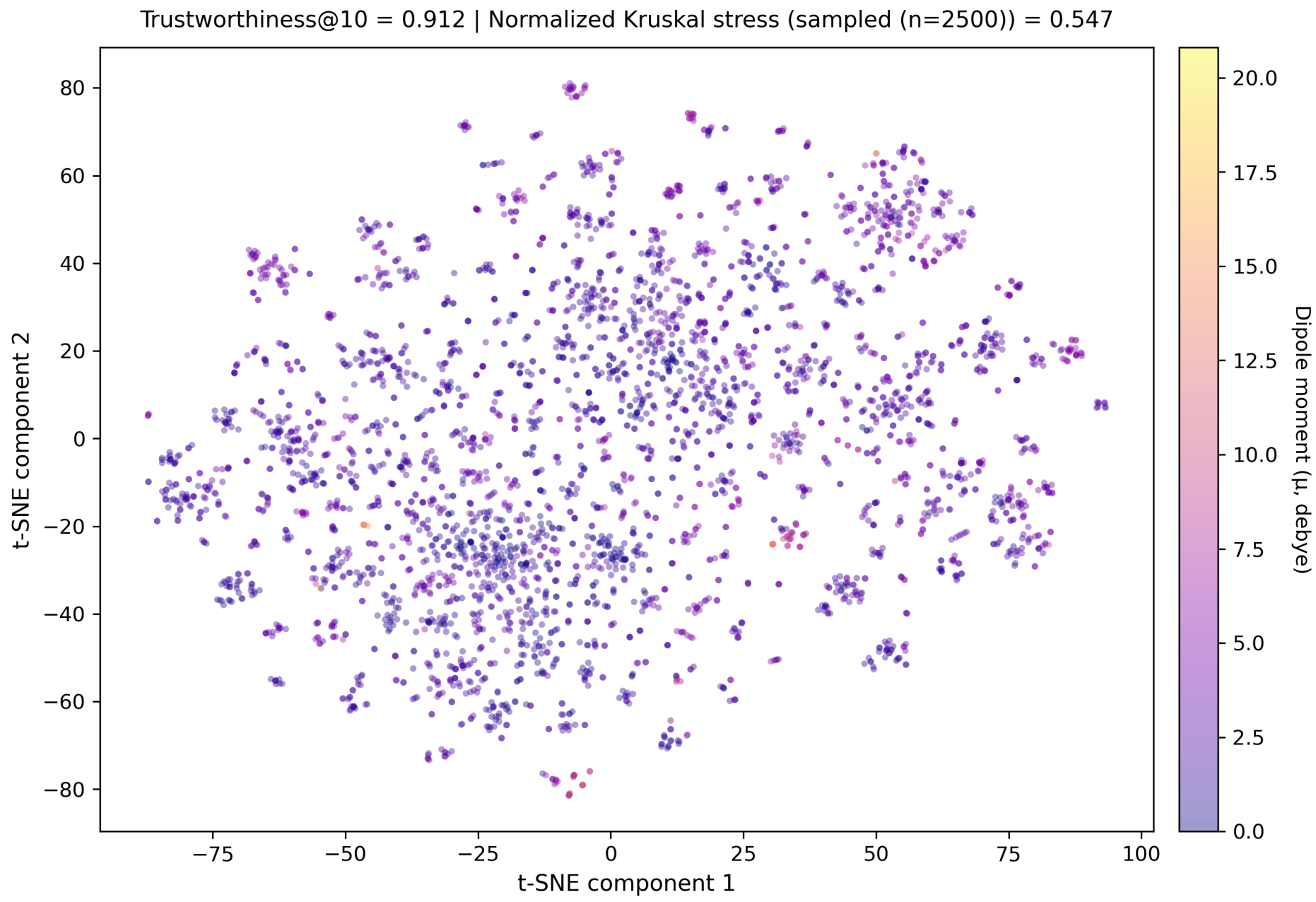

Fig. 10: Two-dimensional t-SNE projection of the final feature space used in the CatBoost model, with points colored by the experimental dipole moment (μ, Debye). The axes correspond to the first and second t-SNE embedding components. Embedding quality diagnostics are reported in the title: Trustworthiness@10 = 0.912 and normalized Kruskal stress (computed on a sampled subset of $n = 2500$ molecules) = 0.547. To improve readability in dense regions, points were rendered with semi-transparency, so local structures should be interpreted as distributional patterns rather than exact point counts.

Figure 10 indicates that the extended descriptor set forms multiple compact islands and semi-connected neighborhoods rather than a single continuous cloud, suggesting that the final feature space is organized into several local structural regimes. The reported Trustworthiness@10 value of 0.912 supports the view that a substantial portion of nearest-neighbor relations from the original high-dimensional space is retained in the two-dimensional embedding, making the map suitable for qualitative interpretation of local geometry. At the same time, the normalized Kruskal stress value of 0.547 indicates that the low-dimensional projection still introduces non-negligible distortion, which is expected when a heterogeneous, high-dimensional molecular representation is compressed into two dimensions. In practical terms, this means that the t-SNE map is most reliable for interpreting local neighborhoods rather than global inter-cluster distances.

Coloring by dipole moment further indicates that μ varies in a heterogeneous manner across the manifold rather than along a single smooth global gradient. High-μ

molecules appear as scattered pockets and isolated points across different neighborhoods, while many local regions still exhibit mixed color intensities. This pattern implies that even within structurally similar neighborhoods, dipole moment can vary meaningfully, likely due to the combined influence of subtle substructure effects, descriptor interactions, and experimental variability. Nevertheless, some local clusters display partial color coherence, indicating that the extended descriptor set does capture neighborhood-level structure—property continuity for subsets of the chemistry space. Overall, the t-SNE map supports a view of the final feature space in which predictive behavior is governed by local, region-specific structure-property relationships rather than by a single global rule, further justifying the use of flexible non-linear learners for dipole-moment prediction.

5.1.3 Feature Relevance Profiling Using ANOVA F-Statistics

Figure 11 presents the univariate feature relevance profile of the final extended descriptor set using ANOVA F-regression scores with respect to the experimental dipole moment. A pronounced ranking gradient is observed, with the three highest-scoring features—gasteiger_multiconf_mean, gasteiger_multiconf_boltzmann, and gasteiger_single—showing substantially larger F-scores than the remaining variables. These are followed by TPSA and NumHAcceptors, after which the distribution declines toward a mixed group of MACCS and Avalon fingerprint features. The dominance of the top-ranked dipole-related descriptors is chemically plausible, since these variables are designed to encode charge-distribution behavior that is directly relevant to dipole moment. At the same time, the strong appearance of TPSA and hydrogen-bond acceptor count among the leading features indicates that polarity-related 2D physicochemical descriptors also contribute a substantial marginal signal. Beyond these top entries, the remainder of the top-25 list is largely populated by fingerprint variables, especially Avalon features together with several MACCS bits, suggesting that substructure-based encodings still provide an important complementary source of structure–property information.

Overall, Figure 11 suggests that the ANOVA ranking is not dominated by a single representation family, but instead reflects a layered relevance pattern in which dipole-related descriptors provide the strongest individual associations, 2D physicochemical descriptors occupy the next most prominent positions, and fingerprint features contribute a broad secondary signal. In terms of feature-group prevalence within the top-ranked set, Avalon and MACCS variables are numerically the most frequent, whereas the very top of the ranking is controlled by the dipole-related and 2D descriptor blocks. This distinction is important for interpretation: the descriptors at the top appear to capture direct physicochemical correlates of dipole moment, while the fingerprints seem to encode more distributed structural cues that may become especially useful when combined in multivariate learners. Because ANOVA F-statistics quantify marginal linear association only, these results should be interpreted as a screening-level relevance profile rather than a full measure of predictive importance. Nevertheless, the ranking clearly supports the inclusion of the extended descriptor set in the final model, as its highest-scoring members contribute the strongest univariate signal observed in the entire feature space.

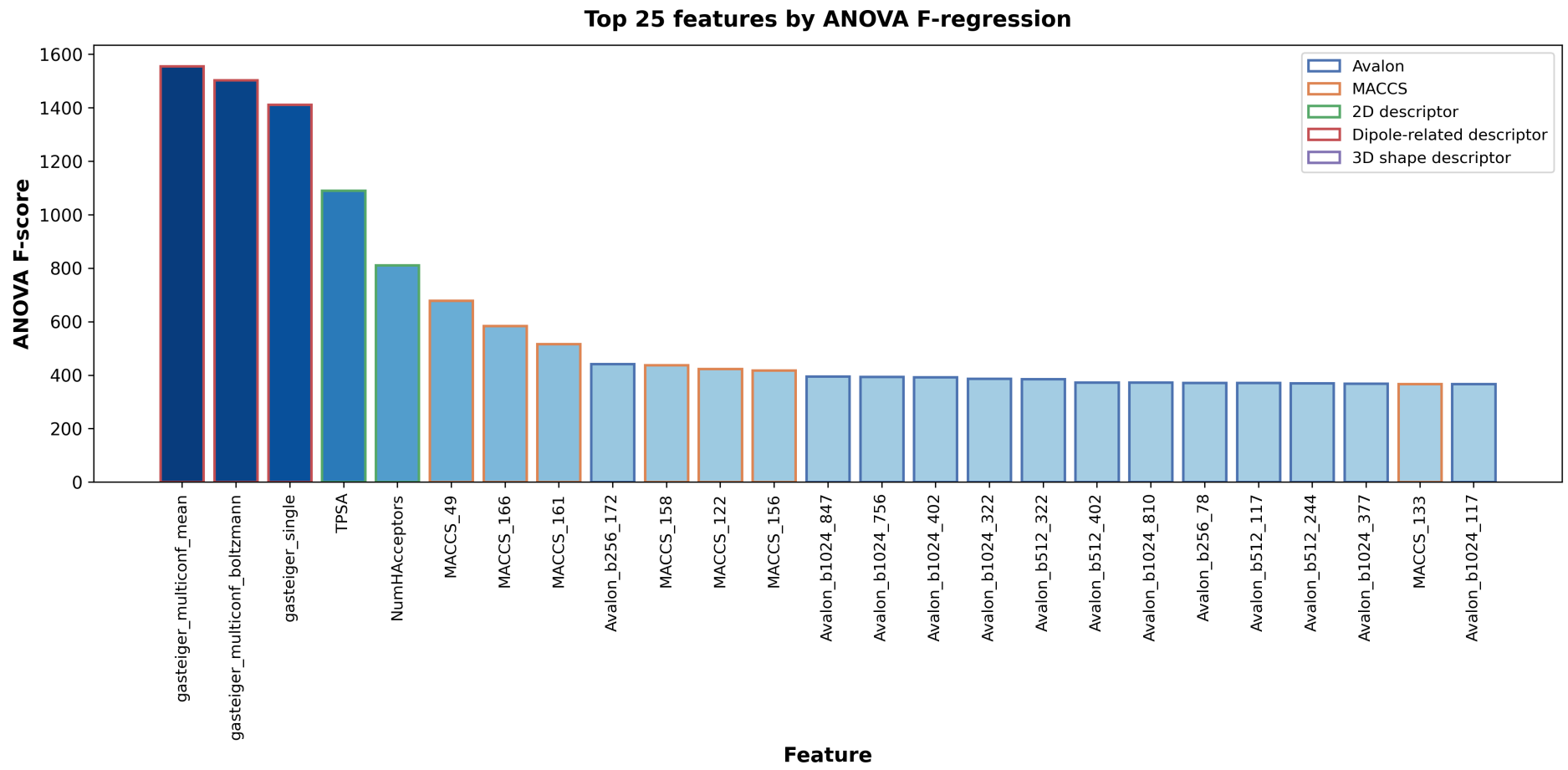

Fig. 11: Top 25 features ranked by ANOVA F-regression score with respect to the experimental dipole moment (μ) for the final extended descriptor set. Bar colors denote feature groups, including Avalon fingerprints, MACCS fingerprints, 2D descriptors, dipole-related descriptors, and 3D shape descriptors. The highest-ranked features are dominated by dipole-related descriptors, followed by polarity-associated 2D descriptors and a broader set of fingerprint-derived variables, indicating that both electronic proxy features and structured 2D representations contribute strongly to the variance associated with μ.

5.2 Cross-Method Comparison of SHAP and LIME Feature Rankings

To compare global and local explanation patterns in the CatBoost model, the most influential features identified by SHAP and LIME were aligned in a single summary table after removing duplicate feature entries. When duplicate rows were present in the raw ranking outputs, only one unique entry per feature was retained.

Table 14 shows a clear area of agreement between the global SHAP analysis and the local LIME analysis. In particular, MACCS_163 appears among the most influential variables in both methods, with a mean absolute SHAP value of 0.456881 (rank 2) and a mean absolute LIME weight of 0.612674 (rank 1). A similar cross-method consistency is observed for gasteiger_multiconf_mean (0.193375; SHAP rank 4 and 0.425378; LIME rank 7), gasteiger_multiconf_boltzmann (0.164963; SHAP rank 5 and 0.278645; LIME rank 10), MACCS_166 (0.057393; SHAP rank 10 and 0.542232; LIME rank 3), MACCS_158 (0.108650; SHAP rank 8 and 0.252536; LIME rank 17), MACCS_41 (0.028713; SHAP rank 17 and 0.261877; LIME rank 14), and TPSA (0.194224; SHAP rank 3 and 0.224735; LIME rank 22). This overlap is important because it indicates that both explanation frameworks repeatedly recover a shared subset of chemically meaningful structural and descriptor-based variables, supporting the stability of the interpretability analysis.

At the same time, Table 14 also reveals a useful methodological distinction between the two explanation modes. Several features are prominent only in SHAP, such as mmff_single (0.968258; rank 1), HeavyAtomCount (0.134904; rank 6), PMI1 (0.110389; rank 7), and n_confs_used (0.070727; rank 9), suggesting that these variables contribute strongly to the model's overall global behavior across the test population. In contrast, LIME highlights several locally recurrent fingerprint conditions that do not appear in the SHAP top-25 list, including MACCS_16 (0.534413; rank 5), MACCS_1 (0.376294; rank 8), Avalon_b1024_637 (0.262552; rank 11), Avalon_b1024_63 (0.262552; rank 12), and MACCS_15 (0.252536; rank 18). Taken together, this pattern suggests that the CatBoost model is governed by both globally important continuous descriptor effects and locally important discrete substructure activations. Accordingly, SHAP and LIME should be viewed as complementary rather than redundant, with SHAP emphasizing population-level attribution structure and LIME exposing molecule-specific decision rules that recur across local neighborhoods.

5.3 Extended Comparative Analysis Across Diverse Regression Families

Table 15 reports an extended benchmarking suite designed to reduce model-selection bias by demonstrating that the observed performance trends are not artifacts of a narrowly chosen baseline set. All regressors were evaluated under the same internal 5-fold cross-validation protocol using the same fingerprint-based tabular input representation composed of MACCS keys and radius-based Avalon fingerprints, and performance is summarized as mean ± standard deviation for R^2, MAE, and RMSE. The inclusion of multiple algorithmic families—gradient-boosted tree learners, bagging/forest ensembles, kernel methods, neural-network baselines, robust/regularized linear models, and similarity-based predictors—provides broad coverage of conventional tabular-learning paradigms and enables cross-family consistency checks.

Across the strongest tabular learners, a clear and reproducible pattern emerges: modern gradient-boosting methods consistently occupy the top tier under identical evaluation conditions. CatBoost achieves the highest mean R^2 among the tested classical models (0.73 ± 0.04) with MAE $= 0.56 \pm 0.04$ and RMSE $= 0.91 \pm 0.15$, closely followed by LightGBM (0.72 ± 0.03) and XGBoost (0.71 ± 0.02). Importantly, the performance differences among these top methods are modest and within overlapping uncertainty ranges, indicating that the results do not depend on a single idiosyncratic model choice; rather, multiple boosting implementations converge to similar performance under the same protocol.

A second consistency signal is observed across bagging and forest ensembles. Bagging decision trees and Random Forest both yield $R^2 \approx 0.71$ with similar MAE and RMSE, suggesting that the dataset contains stable predictive signal that is recoverable by multiple tree-ensemble strategies, not only by the selected primary learner. Conversely, kernel-based regressors (SVR-RBF, NuSVR, Kernel Ridge-RBF) and the MLPRegressor baseline perform systematically worse, with lower R^2 and elevated error magnitudes (e.g., SVR/NuSVR: $R^2 = 0.68 \pm 0.03$, RMSE $= 0.98 \pm 0.13$; MLPRegressor: $R^2 = 0.66 \pm 0.04$, RMSE $= 1.00 \pm 0.13$). These trends support the interpretation that, for the present sparse fingerprint-based molecular representation,

tree-based ensembles provide a better inductive bias than smooth kernel or generic dense-network baselines when trained under comparable conditions.

The similarity-driven k-NN baseline ($k = 5$) performs substantially below the learned tree ensembles ($R^2 = 0.51 \pm 0.05$, MAE $= 0.77 \pm 0.07$, RMSE $= 1.21 \pm 0.17$), reinforcing that nearest-neighbour transfer alone is insufficient to capture the non-linear structure-μ relationships present in the experimental setting. Notably, the comparable performance of Kernel Ridge ($R^2 = 0.51 \pm 0.06$) further indicates that simply imposing smoothness in feature space does not substitute for learning higher-order interactions that are more naturally captured by boosted trees in high-dimensional binary descriptors.

Finally, the spread (standard deviation) values provide an additional robustness indicator. Top-performing boosting/forest models exhibit relatively small variability across folds (typically ± 0.02-± 0.04 in R^2), whereas weaker baselines show larger dispersion (e.g., k-NN and Kernel Ridge: ± 0.05-± 0.06). This pattern suggests that stronger models not only achieve higher mean accuracy but also yield more stable performance with respect to resampling, which is consistent with genuinely informative structure-property signal rather than overfitting to specific partitions.

Overall, the extended benchmarking in Table 15 provides evidence against model-selection bias in the reported study conclusions. Multiple high-capacity tree-ensemble families converge to similar top-tier performance under an identical protocol and the same MACCS + radius-based Avalon fingerprint representation, whereas similarity-only, kernel, and generic neural baselines consistently underperform. This pattern indicates that the observed gains are systematic and attributable to inductive-bias suitability for sparse fingerprint-based molecular encoding rather than to selective reporting. This broad comparison therefore strengthens confidence that the primary learners chosen for the main framework, including CatBoost as the tabular branch, are representative of the strongest conventional tabular approaches for the given experimental setting, and that subsequent hybrid improvements are built upon a robust baseline foundation.

5.4 Local Similarity, Functional Substructure Stratification, and Benchmark Noise

Figure 12 characterizes the evaluation regime by quantifying, for each test compound, the similarity to its closest training analogue (nearest neighbor). This is a more stringent diagnostic than global scaffold counts because it probes the local chemical neighborhood that each test point can leverage. The distribution indicates that only a limited fraction of the test set is nearly duplicated by training examples (100-95%: 12.6%; 95-90%: 9.4%), while the majority lies in intermediate similarity regimes (90-80%: 25.4%; 80-70%: 24.2%; 70-60%: 16.1%). Thus, most test molecules are not evaluated under a trivial "memorization" setting; instead, performance predominantly reflects interpolation across related, but non-identical, neighborhoods and partial extrapolation to less-supported regions.

Dipole moment is particularly sensitive to small structural changes (e.g., heteroatom placement, substitution pattern, conjugation, and conformational preferences). Consequently, when nearest-neighbor similarity drops from the very-high

regime into the 90-70% range, the expected variability in dipole moment within that neighborhood can increase sharply. In practical terms, this means that the benchmark becomes effectively more “noisy” from the model’s perspective: the training set provides fewer near-twins to anchor predictions, and experimental/annotation variability can dominate local signal for many test points. The non-trivial mass in the 60-50% (8.7%) and 50-0% (3.6%) bins further emphasizes the presence of genuinely distant test compounds, where prediction must rely on transferable physico-chemical patterns rather than close analog retrieval.

Taken together, the nearest-neighbor similarity distribution supports the interpretation that the reported test performance is obtained under a realistic generalization regime with meaningful out-of-distribution pressure. In particular, achieving strong accuracy is more demanding here than in datasets where a large share of test compounds has $\geq$95% similarity to training. Therefore, Figure 12 provides supporting evidence that the benchmark is both chemically diverse at the local-neighborhood level and noise-sensitive, and it contextualizes why modest shifts in similarity (even a few percentage points) can lead to disproportionate changes in apparent predictability for dipole moment regression.

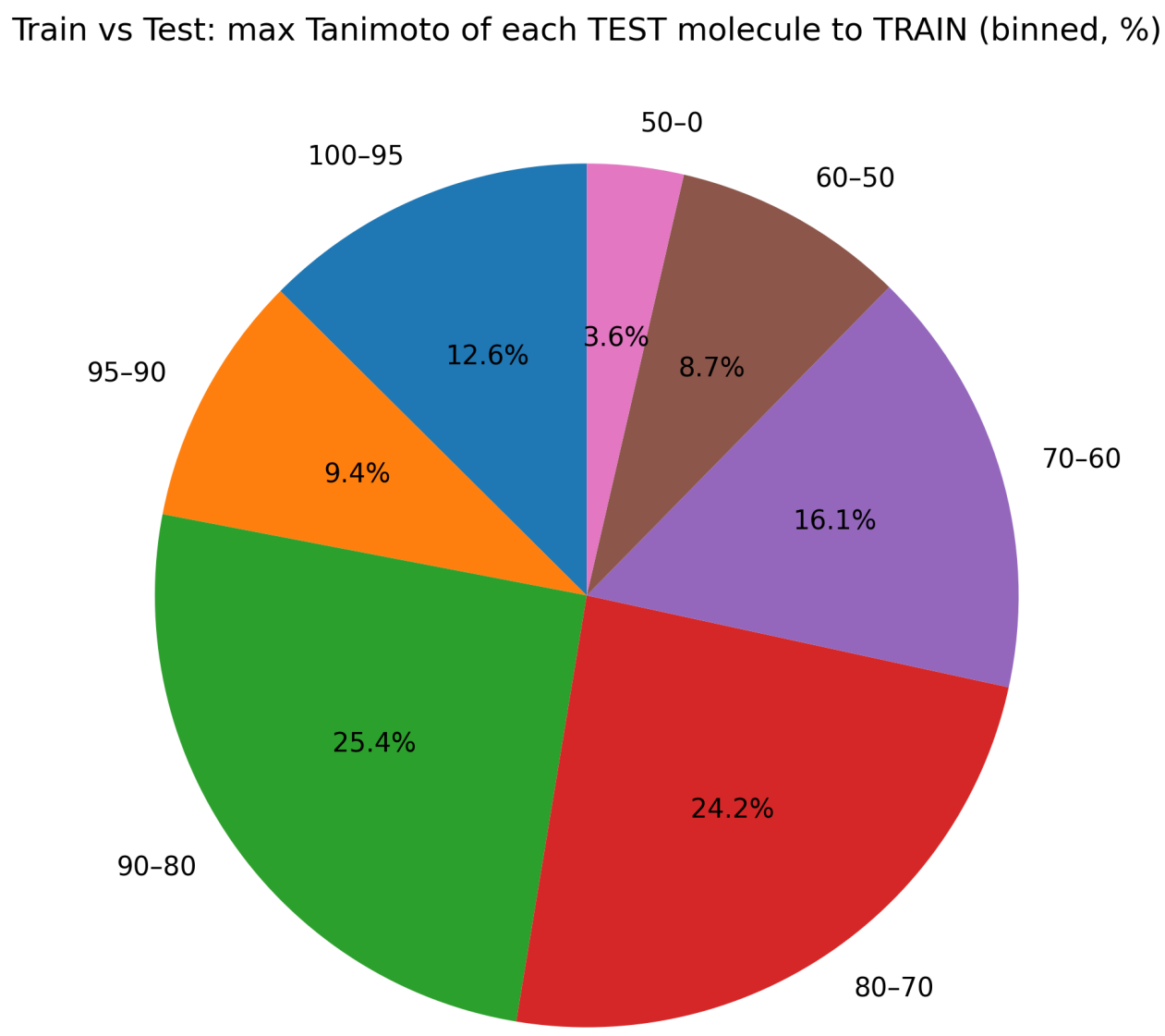

Fig. 12: Train-test nearest-neighbor similarity distribution. For each test molecule, the maximum Tanimoto similarity to any molecule in the training set was computed using the binary fingerprint representation and then binned into percentage intervals (100-95, 95-90, 90-80, 80-70, 70-60, 60-50, and 50-0). The pie chart reports the fraction of test molecules falling into each similarity bin.

Figure 13 refines the coarse 8-class analysis by separating carbonyl- and nitrogen-containing molecules into chemically interpretable subclasses (e.g., carboxylic acids, amides, esters, aldehydes/ketones; and nitriles versus amines). This finer stratification is valuable because dipole moment is not only sensitive to the presence of heteroatoms, but also to the functional context that controls electron withdrawal/donation, resonance, and local charge separation. The distribution shows that the benchmark contains large, chemically distinct polar motifs (notably aldehyde/ketone-type carbonyls, amines, alcohol/ethers, and halogenated compounds), which collectively imply broad coverage of polarity-driving substructures and therefore a wide target range. Such breadth is consistent with a challenging learning problem where multiple mechanistic "sources" of dipole moment coexist (e.g., carbonyl polarization, amine lone-pair effects, halogen-induced inductive shifts, and heteroaromatic ring contributions), rather than a single dominant driver.

As in the main grouping, the train and test panels largely preserve the full-set composition across the 12 classes, suggesting that the split does not introduce a trivial functional-class imbalance that could confound evaluation. At the same time, Figure 13 highlights why the benchmark remains effectively noisy even under stratified distributions. Each class still aggregates considerable intra-class diversity (e.g., "Amine" spans primary/secondary/tertiary amines and diverse substitution patterns; "Aldehyde/Ketone" includes conjugated and non-conjugated systems; "Halogenated" mixes different halogens and substitution positions). Because dipole moment can change substantially with small positional or electronic variations, limited within-subclass replication and heterogeneous micro-environments increase the variance that the model must explain. Notably, rare or absent categories (e.g., nitro being negligible) further emphasize that the dataset is not curated around a narrow functional

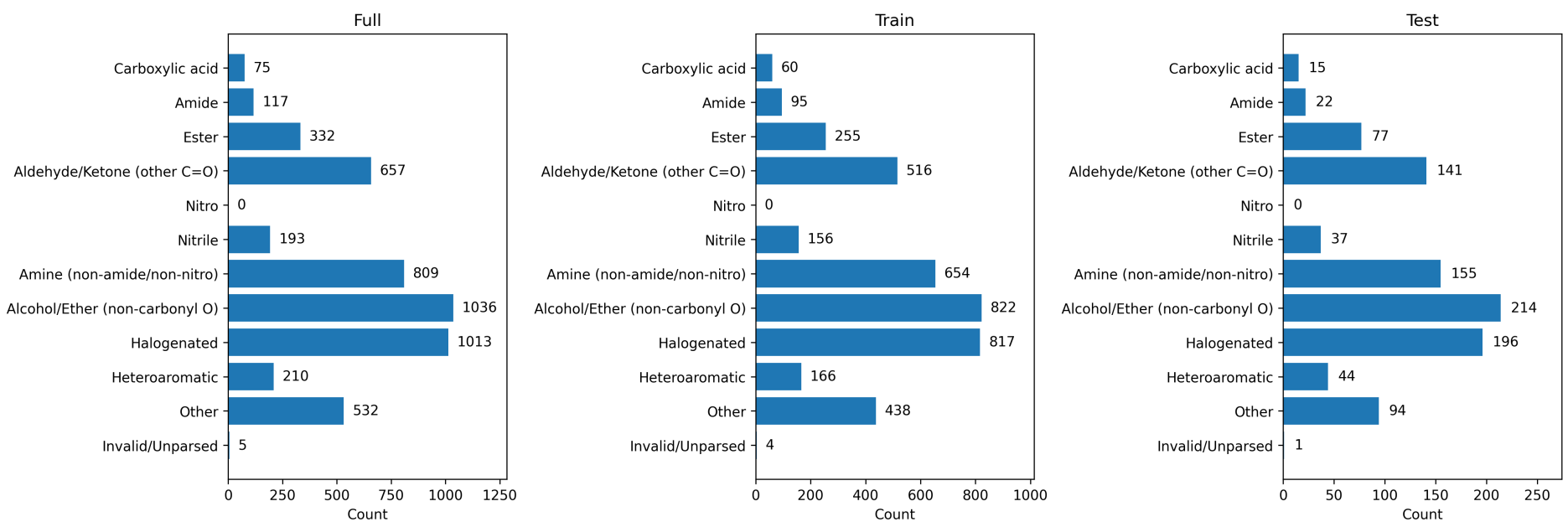

Fig. 13: Chemistry-aware primary-group distribution (supplementary; 12-class) across dataset splits. Horizontal bar charts report the number of molecules assigned to each mutually exclusive functional class—Carboxylic acid, Amide, Ester, Aldehyde/Ketone (other C=O), Nitro, Nitrile, Amine (non-amide/non-nitro), Alcohol/Ether (non-carbonyl O), Halogenated, Heteroaromatic, Other, and Invalid/Unparsed—in the Full dataset (left), Train split (center), and Test split (right). Numeric labels at the end of each bar denote the corresponding counts.

In summary, this section triangulates benchmark difficulty from two complementary perspectives: local neighborhood support and chemistry-aware functional composition. The test-to-train nearest-neighbor analysis (Figure 12) shows that only a minority of test molecules are near-duplicates of training examples, while most fall into intermediate similarity regimes and a non-negligible fraction resides in genuinely low-similarity regions. This directly indicates meaningful out-of-distribution pressure at evaluation time and explains why the benchmark is highly sensitive to small structural differences-a critical consideration for dipole prediction where subtle substitutions can induce disproportionate property changes. In parallel, the 12-class functional stratification (Figure 13) confirms that both training and test splits preserve a broad distribution of polarity-driving motifs (carbonyl subclasses, amines, alcohol/ethers, halogenated and heteroaromatic chemotypes), thereby reducing the likelihood that performance is dominated by trivial class-prevalence shifts. Crucially, the refinement also emphasizes the dominant source of effective noise: each functional bucket remains internally heterogeneous, so local structural and electronic variation (together with experimental variability) limits the attainable signal within many neighborhoods. Collectively, these analyses justify interpreting downstream performance as robust generalization under a chemically diverse, noise-constrained regime rather than an artifact of near-duplicate test compounds or split imbalance.

5.5 Conformer Robustness Analysis for Direct Charge-Based Dipole Proxies

To examine whether the weak performance of direct charge-based dipole approximations could be improved by reducing sensitivity to any single conformer choice, we performed an additional robustness analysis based on RDKit-derived Gasteiger and MMFF partial charges combined with multi-conformer aggregation. For each molecule, four inference schemes were evaluated on the unseen test set: a single representative conformer, arithmetic mean pooling across conformers, median pooling, and Boltzmann-weighted averaging. This analysis was designed to test whether the poor behavior of direct dipole proxies arises primarily from an arbitrary conformer-selection effect or instead reflects a more fundamental limitation of the proxy formulation itself.

The predictive results of these aggregation strategies are summarized in Table 16.

Several observations emerge from Table 16. First, changing the conformer aggregation rule does not materially alter the overall outcome: all direct charge-based variants remain poor predictors of the experimental target, and all test-set R^2 values remain negative. This indicates that the main limitation is not simply the use of a single conformer at inference time. Second, the small numerical differences between aggregation schemes do not reveal a consistent advantage for more elaborate conformer pooling. For the Gasteiger-based estimates, median aggregation gives the least unfavorable result ($R^2 = -0.12$, MAE $= 1.12$), whereas for MMFF-based estimates the single- and median-conformer variants are marginally stronger than the mean and Boltzmann-weighted alternatives. However, these differences are modest and remain far below the performance range of descriptor-based and learned 2D+3D models discussed in the main framework analysis.

From a modeling perspective, this behavior is informative. A direct charge-and-coordinate reconstruction treats the dipole moment as if it were adequately recoverable from a low-level partial-charge assignment applied to approximate conformers. The present results suggest that this assumption is too restrictive for the Stenutz experimental benchmark. The target values in this dataset reflect not only molecular geometry, but also heterogeneous experimental conditions, structural-resolution ambiguity, and residual mismatch between resolved canonical structures and the true molecular states underlying the reported measurements. Under such conditions, simple charge-based proxies appear unable to absorb the broader sources of variation that learned descriptor-based and hybrid models can partially capture through richer structural and statistical representations.

To determine whether these weak results might nevertheless be explained by limited conformer availability or widespread conformer-generation failure, we also recorded practical coverage statistics for the same protocol. These results are summarized in Table 17.

Table 17 shows that the procedure was technically feasible for the majority of molecules, with a success rate of approximately 0.89 in both the training and test sets and with roughly eight usable conformers per successful molecule on average. This is an important control, because it indicates that the poor predictive behavior in Table 16 cannot be attributed simply to insufficient conformational sampling or to large-scale collapse of the conformer-generation workflow. In other words, the method usually succeeded in producing multiple candidate geometries, yet the resulting charge-based dipole estimates still failed to recover meaningful predictive signal.

Taken together, these findings support a more substantive conclusion. The weakness of the direct dipole regime appears to arise not primarily from conformer-selection instability, but from a representational mismatch between simple charge-based proxy calculations and experimentally observed dipole behavior in a heterogeneous real-measurement setting. Even when multiple conformers are generated and aggregated through reasonable statistical schemes, the resulting scalar estimates remain too crude to capture the combined effects of stereochemical ambiguity, state dependence, and experimental variability present in the benchmark. Accordingly, this robustness analysis reinforces the central rationale of the proposed framework: improved performance in experimental dipole prediction is more likely to come from richer learned 2D+3D representations and complementary model fusion than from further refinement of direct low-level charge-based approximations alone.

5.6 Stereochemistry Audit and Its Practical Impact on the Present Representation Pipeline

To clarify how much stereochemical information was actually present and practically propagated through the current dataset-processing workflow, we performed an additional stereochemistry audit on the final curated train–test split. The analysis was designed to address three related questions: first, how frequently explicit stereochemical annotation appears in the benchmark; second, to what extent removal of stereochemical information collapses otherwise distinct molecular identities; and third, whether the fingerprint encodings examined in the present audit are materially

altered by stereochemical stripping. To distinguish between different sources of configurational information, tetrahedral chirality and double-bond stereochemistry were also quantified separately.

The overall stereochemical composition of the dataset is summarized in Table 18.

Several points follow from Table 18. First, explicit stereochemical information is present in the benchmark, but it is not a dominant characteristic of the dataset as a whole. Raw SMILES strings containing explicit stereo tokens account for only 4.75% of the training set and 3.92% of the test set. A broader structure-level inspection indicates that tetrahedral stereochemistry is detectable in a somewhat larger minority of molecules (10.90% in training and 9.64% in test), but explicitly assigned tetrahedral configurations remain rare, comprising only 1.31% and 1.00% of the two splits, respectively. Double-bond stereochemistry is likewise present but limited, affecting 3.44% of the training set and 2.91% of the test set. Taken together, these results indicate that stereochemical variability exists in the benchmark, yet it is concentrated in a relatively small subset of compounds rather than being a pervasive property of the full dataset.

A second observation is that stereochemical stripping causes only limited collapse at the level of unique molecular identities. In the training split, the number of unique stereo-aware structures decreases from 3937 to 3914 after stripping, corresponding to a reduction of 23 distinct structures, whereas in the test split the reduction is only one structure (993 to 992). This pattern suggests that, although some stereochemically distinct forms are indeed merged when stereochemical information is removed, such cases are relatively uncommon in the final curated partition. In practical terms, the benchmark does contain stereo-distinct entries, but the fraction of molecules for which stereochemistry strongly changes identifier-level uniqueness remains modest.

To determine whether these stereochemical distinctions were actually reflected in the fingerprint encodings examined in the present audit, we further compared representations before and after stereochemical removal. The results are summarized in Table 19.

The result in Table 19 is, from a modeling perspective, the most consequential one: stereochemical stripping produced no detectable change in the MACCS or Morgan fingerprints examined here, with zero altered fingerprints in both the training and test splits. This indicates that, within the present audit workflow, the practical representation of stereochemistry at the fingerprint level was effectively negligible. In other words, although a limited number of stereo-distinct structures exist at the molecular-identity level, that distinction was not propagated in a meaningful way into the fingerprint representations examined in this analysis. For this reason, it would be too strong to argue that the 2D identifier-level pipeline retained even partial stereochemical sensitivity in a practically relevant sense.

This finding also helps refine the interpretation of the broader modeling framework. The observed performance gains of the 3D branch should not be interpreted as evidence that the present system explicitly systematically resolves stereochemical identity. Rather, the geometry-aware 3D GNN contributes structural information through conformer-derived molecular graphs in a more general sense, capturing aspects of spatial organization that are not fully available to the tabular branch, but

without constituting a dedicated stereochemistry-aware learning framework. Accordingly, the main limitation is not that stereochemistry is irrelevant to dipole behavior, but that its representation in the current benchmark pipeline is too sparse and insufficiently propagated to support strong stereo-specific claims.

More broadly, the audit suggests that stereochemistry is best viewed here as a localized rather than dataset-defining source of ambiguity. For the majority of compounds, the practical behavior of the current representation pipeline is governed more strongly by general structural encoding choices, conformer approximation, and heterogeneous experimental labeling than by systematic stereo-aware differentiation. This interpretation is important for framing the present study conservatively: the proposed framework can be described as geometry-aware, but it should not be overstated as a rigorous benchmark of stereochemistry-sensitive dipole prediction. Instead, the audit clarifies that any future attempt to study stereo-specific effects more directly would likely require a benchmark with more explicit stereochemical annotation, better propagation of stereo information through identifier-level encodings, and experimentally better-resolved structure-state assignments.

5.7 Computational Cost and Practical Scalability Analysis

To complement predictive performance with a deployment-oriented perspective, we further examined the approximate computational profile of the main components of the proposed framework. This analysis is especially relevant because the framework combines a computationally lightweight tabular branch with a substantially heavier geometry-aware branch, and the practical utility of such a design depends not only on accuracy but also on whether the added 3D signal can be incorporated at an acceptable computational cost. The aim here is therefore not to provide a hardware-independent benchmark, but rather to summarize the relative cost structure of the workflow under a consistent implementation setting. All timings were obtained under CPU execution on Ubuntu using the same fixed train-test protocol and should be interpreted as approximate wall-clock references for the present setup.

The component-level cost summary is reported in Table 20.

Several practical conclusions follow from Table 20. First, the 2D branch is computationally inexpensive throughout the pipeline. Both fingerprint extraction and standard 2D descriptor generation remain fast under CPU execution, and CatBoost training and inference are correspondingly lightweight relative to the rest of the workflow. This makes the tabular branch particularly attractive for large-scale screening, where throughput and robustness are often more important than extracting the full geometric detail of every candidate molecule.

Second, the dominant computational burden arises not from the hybridization step itself, but from the 3D side of the framework. In particular, RDKit-based conformer generation and force-field relaxation represent the principal preprocessing bottleneck, while the 3D GNN introduces the main learning-stage overhead. This distinction is important, because it shows that the additional cost of the geometry-aware branch is concentrated primarily in generating and processing usable 3D molecular structures rather than in the final prediction fusion. In other words, the computational challenge

is largely structural preprocessing and graph-based modeling, not ensemble complexity per se.

Third, the final hybrid remains operationally simple despite combining two representation regimes. Once branch-level predictions have been obtained, the 1:1 linear fusion introduces essentially no additional optimization, meta-learning, or calibration overhead at deployment time. This is a favorable practical property of the present design: the framework gains from complementary 2D and 3D signal integration without requiring a second-stage stacking model or a computationally heavy fusion procedure. From an engineering perspective, this preserves interpretability and simplifies deployment relative to more elaborate ensemble schemes.

Taken together, the cost profile suggests a natural two-tier usage scenario. For rapid prescreening over large molecular sets, the CatBoost branch offers the most favorable speed-to-accuracy balance and can be deployed as a primary ranking model with minimal computational burden. For smaller candidate sets, or for later screening stages in which geometry-sensitive refinement is worthwhile, the 3D branch can be added to improve structural expressiveness at a moderate-to-high extra cost. Under this view, the proposed hybrid framework is practically scalable because it does not require the most expensive components to be used uniformly across all stages of a screening campaign. Instead, it supports a staged deployment logic in which inexpensive 2D screening is followed by optional geometry-aware refinement.

More broadly, this computational pattern is consistent with the conceptual structure of the framework itself. The strongest predictive performance is achieved when 2D and 3D information are combined, but the associated cost increase is highly asymmetric: most of the additional burden is paid before fusion, at the stage of constructing usable 3D inputs. Accordingly, the practical value of the hybrid model lies in offering a controllable tradeoff between speed and structural richness rather than in claiming uniformly low computational cost. In screening-oriented applications, this tradeoff is often preferable to a purely 3D-first strategy, because it preserves a fast baseline pathway while allowing selective use of the more expensive geometry-aware branch when the expected value of additional structural information is highest.

Table 12: Representative case-study molecules used to illustrate per-compound predictive behavior of the proposed framework. The 15 molecules were selected as a fixed random illustrative subset rather than as best-case or worst-case examples.

Name	Dipole moment	CatBoost	3D GNN	Hybrid	Closest model
(E)-2-chlorobenzaldehyde oxime	1.63	1.5154	1.4409	1.4782	CatBoost
cis-3-methylcyclohexanol	1.91	1.6134	1.7041	1.6588	3D GNN
trans-3,3'-dichlorostilbene	1.94	1.5753	1.7995	1.6874	3D GNN
(E)-but-2-enoic acid	2.13	2.2600	2.2764	2.2682	CatBoost
(E)-1-(4-bromophenyl)-3-(1H-pyrrol-2-yl)prop-2-en-1-one	2.52	2.7473	2.6407	2.6940	3D GNN
p-bromophenyldiethylarsine oxide	5.03	5.4454	–	–	CatBoost
(E)-3-furan-2-yl-1-(4-methoxyphenyl)prop-2-en-1-one	3.22	3.4825	3.4850	3.4838	CatBoost
(2E,4E)-1-furan-2-yl-5-phenylpenta-2,4-dien-1-one	3.27	3.3153	3.1520	3.2337	Hybrid
(E)-1,3-bis(1H-pyrrol-2-yl)prop-2-en-1-one	2.93	3.4683	2.9564	3.2124	3D GNN
(E)-1-furan-2-yl-3-(4-methoxyphenyl)prop-2-en-1-one	3.59	3.6974	3.6449	3.6712	3D GNN
(2Z)-penta-2,4-dienenitrile	3.83	3.6298	3.5749	3.6024	CatBoost
(Z)-3-nitrobenzaldehyde oxime	3.95	4.1884	4.0101	4.0993	3D GNN
(E,E)-1-phenyl-5-(2,4,6-trimethoxyphenyl)-1,3-pentanediene-5-one	4.59	4.6179	4.7179	4.6679	CatBoost
(E)-3-(1H-indol-3-yl)-1-naphthalen-1-ylprop-2-en-1-one	5.60	5.5130	5.2004	5.3567	CatBoost
cis-1-tert-butylsulfanyl-2-ethylsulfonylethene	5.90	5.1484	4.7838	4.9661	CatBoost
(1R,4S,5R,6S)-5,6-dicyanobicyclo[2,2,2]oct-2-ene	6.53	4.3109	4.0668	4.1888	CatBoost
pentyl 2-[2-(cyclohexyloxy)ethoxy]ethyl 1,2-benzenedicarboxylate	3.62	3.2573	2.7862	3.0217	CatBoost
3,7,10-trimethyl-2,8,9-trioxa-5-aza-1-borabicyclo[3.3.3]undecane	6.61	4.4036	3.5268	3.9652	CatBoost
9-anthrylidene-2,4,6-trimethylaniline	1.76	1.8379	1.1590	1.4985	CatBoost
2-ethoxy-1,3,2-dioxaphosphorinane	3.05	2.6679	2.9413	2.8046	3D GNN
2-[(4-nitrophenyl)methylidene]propanedinitrile	3.10	4.2168	2.3919	3.3043	Hybrid

Table 13: Stereochemistry relevance and 3D-branch coverage audit for the combined benchmark ($n = 4979$). The analysis was performed on the curated SMILES-based dataset used in the study. Potential tetrahedral stereocenters denote molecules for which RDKit detected at least one potential tetrahedral stereocenter, whereas assigned tetrahedral stereocenters denote molecules with explicitly assigned tetrahedral stereochemistry. Diastereomerism-capable molecules were defined as those containing at least two potential tetrahedral stereocenters. Potential E/Z stereobonds denote molecules with at least one potentially stereogenic double bond. Stereo-relevant molecules were defined as the union of molecules capable of diastereomerism or containing at least one potential E/Z stereobond. Fully specified among stereo-relevant denotes the subset of stereo-relevant molecules for which all relevant tetrahedral and/or E/Z stereochemical elements were explicitly assigned. The 3D-success summary reports the practical coverage of the 3D GNN preprocessing pipeline under the same settings used in the main framework (RDKit ETKDG/ETKDGv3 conformer generation, MMFF94 relaxation when available, UFF fallback disabled, and organic-only filtering).

**Metric**	**Count**	**Percent (%)**
Total molecules	4979	100.00
Valid SMILES	4974	99.90
Molecules with potential tetrahedral stereocenters	530	10.64
Molecules with assigned tetrahedral stereocenters	62	1.25
Molecules capable of diastereomerism ($\geq$ 2 potential tetrahedral stereocenters)	243	4.88
Molecules with potential E/Z stereobonds	166	3.33
Stereo-relevant molecules (($\geq$ 2 tetrahedral) or ($\geq$ 1 E/Z))	409	8.21
Fully specified among stereo-relevant molecules	221	54.03
Stereo-relevant molecules with valid 3D preprocessing	407/409	99.51
Non-stereo-relevant molecules with valid 3D preprocessing	4173/4570	91.31
All molecules with valid 3D preprocessing	4580/4979	91.99

Table 14: Comparison of unique feature rankings obtained from SHAP and LIME for the CatBoost-based tabular model. SHAP values reflect global contribution magnitudes across the test set, whereas LIME weights summarize average local contributions across the explained samples. Duplicate rows in the raw ranking outputs were removed prior to constructing this table. Abbreviations: SHAP-M = mean absolute SHAP value; SHAP-R = SHAP rank; LIME-M = mean absolute LIME weight; LIME-R = LIME rank.

**Feature**	**SHAP-M**	**SHAP-R**	**LIME-M**	**LIME-R**
mmff_single	0.968258	1	–	–
MACCS_163	0.456881	2	0.612674	1
TPSA	0.194224	3	0.224735	22
gasteiger_multiconf_mean	0.193375	4	0.425378	7
gasteiger_multiconf_boltzmann	0.164963	5	0.278645	10
HeavyAtomCount	0.134904	6	–	–
PMI1	0.110389	7	–	–
MACCS_158	0.108650	8	0.252536	17
n_confs_used	0.070727	9	–	–
MACCS_166	0.057393	10	0.542232	3
gasteiger_single	0.054899	11	–	–
mmff_multiconf_mean	0.046646	12	–	–
MACCS_161	0.046078	13	–	–
mmff_multiconf_boltzmann	0.043972	14	–	–
MACCS_148	0.034592	15	–	–
InertialShapeFactor	0.029507	16	–	–
MACCS_41	0.028713	17	0.261877	14
RadiusOfGyration	0.024890	18	–	–
PMI3	0.023105	19	–	–
Avalon_b512_105	0.022186	20	–	–
FractionCSP3	0.022132	21	–	–
MACCS_134	0.020638	22	–	–
MACCS_145	0.020271	23	–	–
MolLogP	0.019966	24	–	–
MolWt	0.019547	25	–	–
Avalon_b1024_637	–	–	0.262552	11
Avalon_b1024_63	–	–	0.262552	12
MACCS_4	–	–	0.261877	13
MACCS_1	–	–	0.376294	8
MACCS_16	–	–	0.534413	5
MACCS_15	–	–	0.252536	18
Avalon_b512_179	–	–	0.231484	21
MACCS_30	–	–	0.222787	23
MACCS_3	–	–	0.222787	24

Table 15: Supporting benchmark of additional regression models evaluated under the same internal cross-validation protocol. All models were trained on the same fingerprint-based tabular representation composed of MACCS keys and radius-based Avalon fingerprints. For each algorithm, the table reports the mean ± standard deviation across folds for R^2, mean absolute error (MAE), and root mean squared error (RMSE). Models include gradient-boosting methods, bagging/forest ensembles, support vector regressors, neural-network baselines, linear/robust regressors, and similarity-based baselines (k-NN, $k = 5$), enabling a broad comparison of conventional tabular-learning approaches.

Models	R^2 with SD	MAE with SD	RMSE with SD
CatBoost	0.73 ± 0.04	0.56 ± 0.04	0.91 ± 0.15
LightGBM (LGBMRegressor)	0.72 ± 0.03	0.56 ± 0.03	0.91 ± 0.13
XGBoost (XGBRegressor)	0.71 ± 0.02	0.55 ± 0.03	0.92 ± 0.12
Bagging (Decision Tree)	0.71 ± 0.03	0.56 ± 0.04	0.93 ± 0.14
Histogram-Based Gradient Boosting (HistGradientBoostingRegressor)	0.71 ± 0.04	0.59 ± 0.04	0.93 ± 0.15
Random Forest	0.71 ± 0.03	0.56 ± 0.04	0.93 ± 0.13
Support Vector Regression (RBF kernel)	0.68 ± 0.03	0.63 ± 0.03	0.98 ± 0.13
Nu-Support Vector Regression (NuSVR)	0.68 ± 0.03	0.63 ± 0.03	0.98 ± 0.13
Multilayer Perceptron (MLPRegressor)	0.66 ± 0.04	0.64 ± 0.05	1.00 ± 0.13
Gradient Boosting Regression Trees (GradientBoostingRegressor)	0.61 ± 0.02	0.75 ± 0.04	1.08 ± 0.13
Bayesian Ridge Regression	0.61 ± 0.04	0.75 ± 0.04	1.08 ± 0.15
Stochastic Gradient Descent Regressor (SGDRegressor)	0.58 ± 0.03	0.78 ± 0.04	1.12 ± 0.13
Huber Regressor	0.57 ± 0.05	0.75 ± 0.06	1.13 ± 0.16
k-Nearest Neighbors Regressor (k=5)	0.51 ± 0.05	0.77 ± 0.07	1.21 ± 0.17
Kernel Ridge Regression (RBF kernel)	0.51 ± 0.06	0.78 ± 0.05	1.21 ± 0.16

Table 16: Test-set conformer robustness analysis for direct charge-based dipole proxies. Results are reported for molecules with successful conformer generation and downstream charge-based dipole estimation.

Method	Test R^2	Test MAE	Test RMSE
Gasteiger single	-0.18	1.17	1.77
Gasteiger mean	-0.21	1.11	1.79
Gasteiger median	-0.12	1.12	1.72
Gasteiger Boltzmann	-0.23	1.14	1.80
MMFF single	-0.05	1.00	1.67
MMFF mean	-0.08	0.94	1.69
MMFF median	-0.05	0.97	1.66
MMFF Boltzmann	-0.07	0.95	1.68

Table 17: Conformer-generation success statistics for the direct charge-based robustness analysis.

Split	n_{total}	n_{success}	Success rate	Mean confs	Median confs	Range
Train	3983	3543	0.89	8.08	5.00	1–20
Test	996	885	0.89	8.20	5.00	1–20

Table 18: Stereochemistry audit of the final curated dataset. Percentages are reported relative to the number of labeled molecules in each split.

Quantity	Train	Test
Total molecules	3983	996
Raw SMILES with stereo tokens	189 (4.75%)	39 (3.92%)
Any tetrahedral stereochemistry	434 (10.90%)	96 (9.64%)
Assigned tetrahedral stereochemistry	52 (1.31%)	10 (1.00%)
Any double-bond stereochemistry	137 (3.44%)	29 (2.91%)
Assigned double-bond stereochemistry	137 (3.44%)	29 (2.91%)
Unique stereo-aware structures	3937	993
Unique stereo-stripped structures	3914	992
Collapsed unique structures after stripping	23	1

Table 19: Effect of stereochemical stripping on the fingerprint representations used in the present audit. Counts refer to molecules whose fingerprints changed after stereochemistry removal.

Split	MACCS diff count	MACCS diff (%)	Morgan diff count	Morgan diff (%)
Train	0	0.00	0	0.00
Test	0	0.00	0	0.00

Table 20: Approximate computational cost of the main components of the proposed framework under CPU execution on Ubuntu. Reported times are implementation-level wall-clock estimates for the setup and are intended for relative comparison rather than strict cross-hardware benchmarking. Abbreviations: FP = MACCS + Avalon fingerprint generation; 2D-Desc = basic 2D descriptor generation; 3D-Desc = RDKit-derived dipole proxies and 3D shape descriptors; ConfGen = 3D conformer generation using ETKDG/ETKDGv3 with MMFF94 relaxation when available (UFF fallback disabled by default); CB-FP = CatBoost fingerprint branch; CB-Ext = CatBoost extended tabular branch; GNN = 3D graph neural network; Hybrid = final linear 1:1 fusion.

**Component**	**Approx. Time**	**CPU/Memory**	**Notes**
FP	15–45 s	Low	RDKit-based 2D fingerprint extraction
2D-Desc	20–60 s	Low	Standard tabular molecular descriptors
3D-Desc	15–45 min	Moderate–High	Requires successful 3D embedding and optimization
ConfGen	30–120 min	High	Main RDKit-side preprocessing bottleneck
CB-FP	1–5 min	Moderate	CPU-friendly tabular learner
CB-Ext	2–8 min	Moderate	Fingerprint + descriptor augmentation
CB inference	< 0.01 s/mol	Very Low	Suitable for high-throughput use
GNN train	30–120 min	High	Main learning-stage bottleneck
GNN inference	0.02–0.20 s/mol	Moderate	Depends on graph size and conformer availability
Hybrid	Negligible	Very Low	Arithmetic averaging only
